# Fluid Layered Ferroelectrics with Global C_∞v_ Symmetry

**DOI:** 10.1002/advs.202202048

**Published:** 2022-07-22

**Authors:** Hirotsugu Kikuchi, Hiroyuki Matsukizono, Koki Iwamatsu, Sota Endo, Shizuka Anan, Yasushi Okumura

**Affiliations:** ^1^ Kyushu University Institute for Materials Chemistry and Engineering 6‐1 Kasuga‐Koen Kasuga Fukuoka 816‐8580 Japan; ^2^ Kyushu University Interdisciplinary Graduate School of Engineering Sciences 6‐1 Kasuga‐Koen Kasuga Fukuoka 816‐8580 Japan

**Keywords:** ferroelectric liquid crystals, memory effect, second harmonic generation, smectic phases

## Abstract

Ferroelectricity in fluid materials, which allows free rotation of molecules, is an unusual phenomenon raising cutting‐edge questions in science. Conventional ferroelectric liquid crystals have been found in phases with low symmetry that permit the presence of spontaneous polarization. Recently, the discovery of ferroelectricity with high symmetry in the nematic phase has attracted considerable attention. However, the physical mechanism and molecular origin of ferroelectricity are poorly understood and a large domain of macroscopically oriented spontaneous polarization is difficult to fabricate in the ferroelectric nematic phase. This study reports new fluid layered ferroelectrics with the C_∞v_ symmetry in which nearly complete orientation of the spontaneous polarization remains stable under zero electric field without any orientation treatment. These ferroelectrics are obtained by simplifying the molecular structure of a compound with a known ferroelectric nematic phase, although the simplification reduced the dipole moment. The results provide useful insights into the mechanism of ferroelectricity due to dipole–dipole interactions in molecular assemblies. The new ferroelectric materials are promising for a wide range of applications as soft ferroelectrics.

## Graphical abstract

Novel fluid layered ferroelectrics with the C_∞v_ symmetry, ferroelectric smectic A phases, are developed by simplifying the molecular structure of a compound which is known to exhibit a ferroelectric nematic phase. Nearly complete orientation of the spontaneous polarization remains stable under zero electric field without any orientation treatment in the ferroelectric smectic A phases.

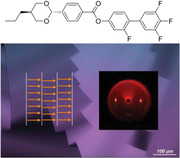

## 1 Introduction

Ferroelectrics are materials in which electrical, mechanical, and thermal properties are mutually coupled, resulting in various attractive and valuable functionalities. Such materials can be used in a wide range of applications, such as in capacitors, memories, sensors, optical devices, actuators, and energy‐conversion devices, and are potentially applicable to other innovative technologies.^[^ ^1^ ^]^ One requisite of a ferroelectric material is spontaneous polarization (P_S_). The crystal symmetries that can generate P_S_ belong to ten point groups: C_1_, C_2_, C_1h_, C_2v_, C_4_, C_4v_, C_3_, C_3v_, C_6_, and C_6v_. In liquid crystals, ferroelectricity has been found in a chiral smectic C phase with a C_2_ symmetry,^[^ ^2^ ^]^ whereas bent‐core smectic liquid crystals exhibit a C_2_ or C_2v_ symmetry.^[^ ^3^ ^]^ In these conventional ferroelectric liquid crystal phases, P_S_ is directed along the lateral face of the molecule and parallel to the smectic layer. In 2017, Mandle et al. found a new nematic phase that differs from the ordinary nematic (N) phase in a compound called RM734.^[^ ^4^, ^5^ ^]^ Mertelj et al. later showed that the low‐temperature N phase of RM734 has a lower symmetry than the ordinary uniaxial N phase.^[^ ^6^ ^]^ In 2017, the year in which Mandle et al. first reported RM734, our research group reported another new nematic phase, that is, the MP phase, in a compound (DIO) with a dioxane group.^[^ ^7^ ^]^ The MP phase exhibits ferroelectric characteristics such as anomalously large dielectric permittivity, large P_S_ in the longitudinal direction of the molecule, electric displacement–electric field (*D*–*E*) hysteresis, second harmonic generation (SHG) activity, and SHG interference reversal due to polarization reversal.^[^ ^7^, ^8^ ^]^ These two new nematic phases of RM734 and DIO, which were observed independently in different substances, have been identified as the ferroelectric nematic (N_F_) phase.^[^ ^9^, ^10^, ^11^ ^]^ The discovery of the N_F_ phase has attracted considerable interest in the materials science field.^[^ ^12^ ^]^ However, the physical mechanism and molecular origin of such ferroelectric phases are poorly understood. The N_F_ phase distinctively exhibits C_∞v_ symmetry, which describes the symmetry of a cone shape and is not included in the abovementioned ten point groups. In reality, however, the orientation of the director in the N_F_ phase exhibits spontaneous distortion, making the global C_∞v_ symmetry difficult to maintain. The textures produced by this distortion have been observed in some studies to be complex, grainy Schlieren, or broken Schlieren.^[^ ^4^, ^7^ ^]^ Without external assistance such as electric fields or surface anchoring, eliminating this distortion to achieve a large‐size domain oriented in a single direction is a difficult task.^[^ ^13^ ^]^ Herein, we present a ferroelectric smectic A phase, a 2D liquid and a 1D crystal, exhibiting C_∞v_ symmetry and P_S_ in the direction of the director (i.e., longitudinal ferroelectricity). This phase is realized by simplifying the molecular structure of the abovementioned DIO compound. Its relatively robust layered structure maintains a large‐size polar domain even in the absence of an external field, thus retaining the global C_∞v_ symmetry in a self‐sustaining manner. Because DIO and the molecules exhibiting this ferroelectric smectic A phase have similar molecular structures, our findings can help elucidate the molecular origin of longitudinal ferroelectricity, which has not yet been clarified.

## 2 Results and Discussion

### 2.1 Molecular Structure and Phase Behavior of Liquid Crystal Compounds with a Dioxane Ring

**Figure 1:**
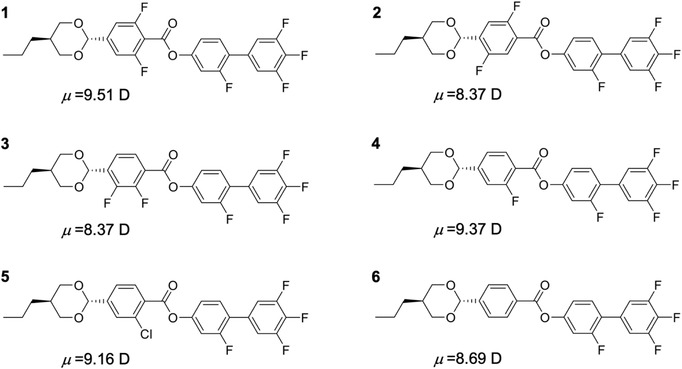
Chemical structures of the liquid crystal compounds investigated in this study. Compound **1** was provided by JNC Petrochemical Corporation. Compounds **2**–**6** were synthesized in our laboratory (see Section S1, Supporting Information, for details).

**Figure** **1** shows the chemical structures of the liquid crystal compounds used in this study. Compound **1** (DIO) has been described in our previous study.^[^ ^7^ ^]^ We newly synthesized compounds **2**–**6** with various substituents differing from those of **1** and compared their properties. We focused on the substituents of the phenyl group directly connected to the dioxane group, that is, the third phenyl group from the right end, hereafter referred to as the “third phenyl group.” Section S1, Supporting Information, describes the synthetic reaction schemes and molecular characterizations of **2**–**6**. The phase‐transition temperatures of various compounds (top part of **Table** **1**) were evaluated using differential scanning calorimetry (DSC). The DSC curves of **2**–**6** are provided in Section S2, Supporting Information. Further, the phase types of these compounds were comprehensively evaluated based on polarized optical microscopy (POM), wide‐angle and small‐angle X‐ray diffraction (WAXD and SAXD, respectively), dielectric permittivity, *D*–*E* hysteresis, and SHG activity. The dielectric permittivity *ɛ*′(∥) was measured using a cell with vertical surface‐alignment treatment. The *ɛ*′(∥) is not necessarily identical to the dielectric permittivity parallel to the director, commonly denoted as *ɛ*′_∥_, because in the case of ferroelectric phases, the dielectric permittivity may be underestimated as ferroelectric phases do not always follow the surface orientation treatment.^[^ ^7^ ^]^ The dielectric relaxation strength *δɛ*(∥) was derived from a Cole–Cole plot based on the frequency dependence of *ɛ*′(∥). Because the dielectric permittivity depends on the measurement frequency, the polarization phenomenon of interest must be analyzed by measuring the dielectric permittivity at a frequency sufficiently lower than the polarization relaxation frequency. However, at low frequencies, electrode polarization due to ion currents can be superimposed on the dielectric permittivity. Therefore, when the ion current cannot be ignored, the relaxation strength of the polarization phenomenon of interest should be evaluated. In the present study, the relaxation strengths were used for evaluating dielectric phenomena in **4** and **6**, which exhibit ferroelectricity. The SHG was regarded as active (A) when the intensity of any phase was greater than or equal to that of a Y‐cut quartz plate measured using the same system; otherwise, it was regarded as inactive (IA).

**Table 1:** Phase‐transition temperatures (top), observed maximum dielectric permittivities, second harmonic generation (SHG) activities, and calculated dipole moments (bottom) of all the compounds

No.^a)^	Phase‐transition temperature [°C]^b)^														
	Cr		SmX_F_		SmA_F_		SmA_F′_		N_F_		N_X_		N		Iso
**1**	•	96							(•	69	•	85)	•	174	•
**2**	•	123											•	196	•
**3**	•	126											•	180	•
**4**	•	107	(•	85	•	106)					•	115	•	207	•
**5**	•	72											•	137	•
**6**	•	129			•	146	•	158					•	231	•

No.^a)^	Max. value of *ε*′(∥)/10^3^ ^c)^	SHG^d)^	*μ* [D]^e)^	*β* [°]^f)^	*µ*_l_ [D] along axis^g)^
**1**	10 at 55°C, 1 kHz	A	9.51	9.42	9.38
**2**	0.0072 at 90 °C, 1 kHz	IA	8.37	13.70	8.13
**3**	0.0086 at 120 °C, 10 kHz	IA	8.37	7.28	8.31
**4**	7.1 at 84 °C, 100 Hz	A	9.37	3.99	9.34
**5**	0.026 at 60 °C, 100 Hz	IA	9.16	9.83	9.02
**6**	4.9 at 148 °C, 100 Hz	A	8.69	10.61	8.54

Because all these compounds can chemically transform or degrade at temperatures above 120 °C, the high‐temperature measurements were performed as quickly as possible.

^a)^ Compound numbers **1**–**6** correspond to the compounds presented in Figure 1

^b)^ Temperatures in parentheses (—) denote monotropic transition temperatures detected using DSC at the descending temperature when the temperature exceeded each melting point. The dots in the table indicate that the compound exhibits the corresponding phase. It is difficult to determine whether the SmA_F_s of **4** and **6** are identical using only our measurements

^c)^ The maximum (Max.) value of *ε*'(∥) represents the maximum dielectric permittivity, an important criterion for determining the presence or absence of ferroelectricity in the sample. The data on the dielectric permittivities of **2**, **3**, and **5 **are given in Figure S4‐1, Supporting Information

^d)^ The “A” and “IA” entries in the second harmonic generation (SHG) column denote active and inactive, respectively. A sample was labeled “A” if any of its phases were active; otherwise, it was labeled “IA.” The ferroelectric phase must be active because it has a noncentrosymmetric structure

^e–g)^ The symbols *μ*, *β*, and *µ*_l_ represent the vector sum of the dipole moments of the whole molecule, the angle between *μ* and the long axis of the molecule, and the component of *μ* along the long axis of the molecule, respectively, calculated using density functional theory with the B3LYP/6‐31+G(2d,p) basis function. The symbol D (Debye) is a unit of electric dipole moment, defined as 1 × 10^−18^ statcoulomb‐centimeters.

### 2.2 Phase Structures and Ferroelectric Properties of Compound **4**

**Figure 2:**
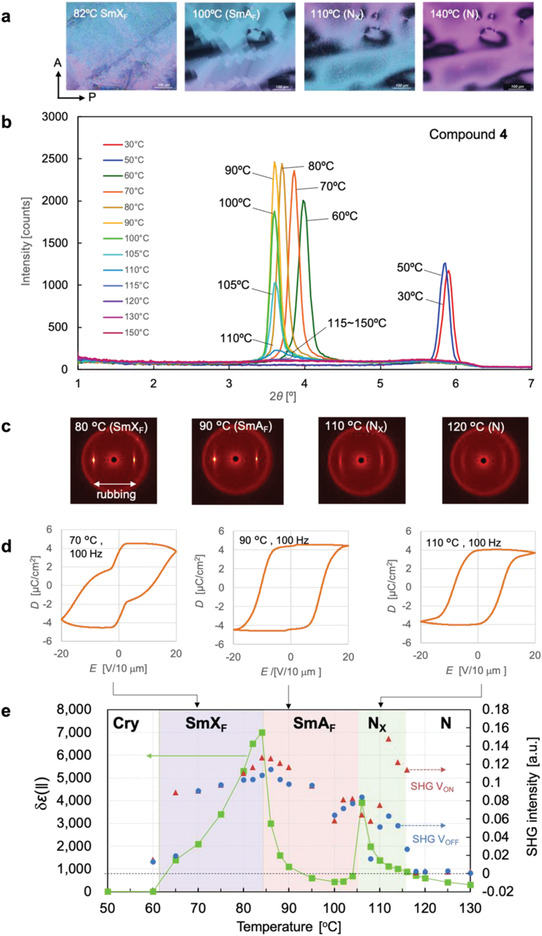
Experimental results of liquid crystal structures and main physical properties of compound **4**. a) Polarizing optical microscopy (POM) images observed on cooling. The POM image at 82 °C is not directly cooled as that at 100 °C but after an orientation change by the in‐plane electric field in the SmA_F_ phase. The substrate surfaces of the cells were coated with poly(methyl methacrylate) (PMMA) to allow free orientation in the plane. The scale bar in each image represents 100 µm. b) Small‐angle X‐ray diffraction profiles measured at various temperatures during the cooling process. c) 2D SAXD images of samples oriented by rubbing in the direction parallel to the equatorial direction. d) Electric displacement–electric field (*D*–*E*) hysteresis curves at 70 °C (SmX_F_), 90 °C (SmA_F_), and 110 °C (N_X_). The voltage was applied parallel to the director. e) Temperature dependences of dielectric permittivity (green squares) and SHG intensities during application and removal of a voltage (red triangles and blue circles, respectively) during the cooling process. Light polarized parallel to the director was incident on the sample for SHG measurements.

**Figures** **2**a and 2b show the temperature dependences of the POM images and SAXD profiles, respectively, based on the cooling process of **4** due to phase monotropicity. The POM image at 140 °C shows the typical Schlieren texture of the N phase. The POM observations in the homeotropic and homogeneous‐orientation cells (Figures S3‐1 and S3‐2, Supporting Information) are also consistent with this phase being N. Upon phase transition at 116 °C, the Schlieren texture became grainy, as shown in the image taken at 110 °C (Figure 2a). In this phase, no sharp diffraction peaks were observed in either the SAXD or WAXD spectra (Figure S5‐1, Supporting Information). Thus, no layered structure or other long‐range translational order existed in this phase. As shown in Figure 2d,e, the *δɛ*(∥) of this phase reached 4000 (as additional data, Figure S4‐2, Supporting Information, displays the frequency dispersion of the dielectric permittivity at each temperature and Figure S4‐4, Supporting Information, plots the temperature dependence of the dielectric permittivity at different frequencies), the *D*–*E* hysteresis typically observed in ferroelectrics and SHG activity were also observed. Because no N_F_‐specific polydomain structure with defect lines was observed in the texture at 110 °C (Figure 2a), we attribute this phase to an unknown nematic phase N_X_ that may differ from the N_X_ phases of other compounds. The comparison of this N_X_ phase with the M2 phase of ref. [7] is of interest, however, it is challenging at this stage to judge whether these two phases are identical or not. Not labeling it as N_F_ does not mean that it is not ferroelectric. The N_X_ phase of compound **4** exhibits clearly ferroelectricity in terms of physical properties, suggesting that it might be an N_F_ phase with domains so finely divided that they cannot be detected with an optical microscope. Upon further cooling and phase transition at 106 °C, the texture suddenly changed to an angular mosaic‐like texture (see the POM image at 100 °C in Figure 2a). Consistent with the temperature dependence of the SAXD profiles (Figure 2b), sharp peaks appeared at 2*θ* = 3.6–4.0° over temperatures ranging from <110 °C to the crystallization temperature (≈50 °C). Therefore, the phases occurring over this temperature range can be identified as smectic phases. In the phase observed at 85–106 °C, the SAXD peaks were almost equally spaced at 2.45 nm, close to the single‐molecule length of **4** (2.25 nm; Section S6, Supporting Information). In addition, no sharp diffraction peaks were observed in the WAXD spectrum at 2*θ* ≈20°, the region corresponding to the lateral intermolecular distance (Figure S5‐1a, Supporting Information). The 2D SAXD image of this phase in an oriented sample sandwiched between rubbed polyimide films showed sharp diffraction spots at 90 °C (Figure 2c). As the diffraction spots appear on the equator parallel to the rubbing direction and the director is oriented parallel to the rubbing direction in this phase (Figure S5‐3, Supporting Information), the plane of the smectic layer is clearly perpendicular to the director. Based on these results, the phase in the 85–106 °C range was identified as the smectic A phase (SmA). In general, higher‐order diffraction appears in lamellar structures as the ratio of the magnitudes of wavenumber vectors is 1:2:3….; however, no higher‐order diffraction was observed in the XRD measurements performed in this study. According to paracrystal theory, higher‐order diffraction is weakened if a second type of disorder exists in the structure^[^ ^14^ ^]^ and disappears when the electron density distribution forming the periodic structure is sinusoidal. These effects may explain the lack of higher‐order diffraction in the smectic A phase of the present study. In addition to producing sharp diffraction peaks in the SAXD spectra, this phase exhibited a large dielectric constant, *D*–*E* hysteresis, and SHG activity, confirming ferroelectricity. Because polarization reversal occurred parallel to the director and SHG was active when light was polarized parallel to the director, this ferroelectricity was identified as longitudinal. Hereafter, we refer to this phase as SmA_F_. The SmA_F_ phase was uniformly and homogeneously oriented in the rubbed cells (Figure S3‐2, Supporting Information) but showed birefringence (albeit with small retardation) texture in the cell with a vertically oriented surface treatment (Figure S3‐1, Supporting Information). It is assumed that the directors did not follow the surface vertical orientation because the orientation change depolarized the generated P_S_, as observed in N_F_.^[^ ^7^ ^]^ At temperatures below 85 °C, the spacing calculated from the SAXD peaks decreased with decreasing temperature and eventually became smaller than the molecular length, suggesting the possibility of another type of smectic phase, which has not yet been identified. The temperature‐dependent diffraction angles and d‐spacings of this phase are shown in Figure S5‐2, Supporting Information. As ferroelectric properties were also observed in this phase, it is here denoted as SmX_F_. Notably, the *D*–*E* hysteresis in the SmX_F_ phase (Figure 2d, 70 °C) presents an antiferroelectric profile instead of the simple parallelogram of a typical ferroelectric phase.

### 2.3 Phase Structures and Ferroelectric Properties of Compound **6**

**Figure 3:**
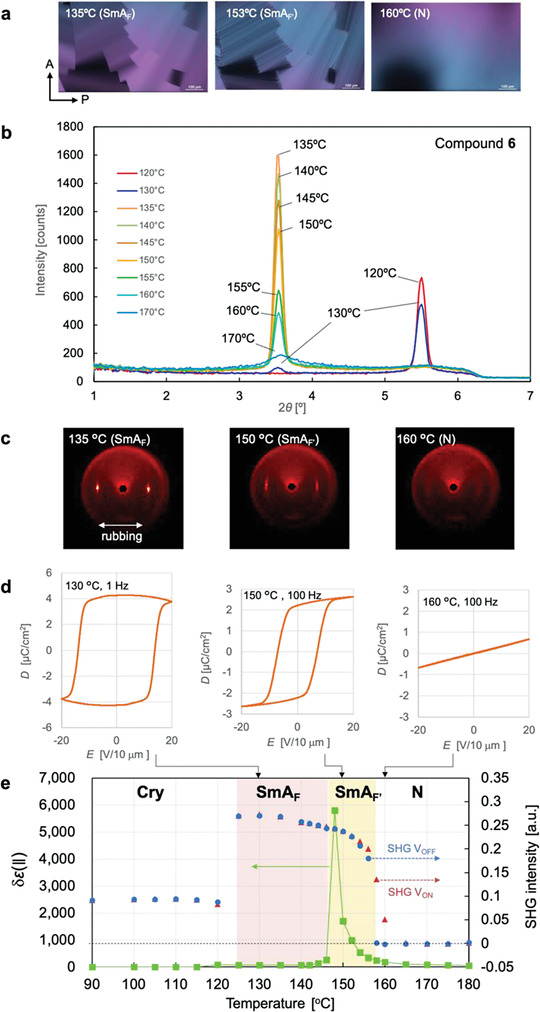
Experimental results of liquid crystal structures and main physical properties of compound **6**. a) POM images observed on heating: 135 °C (SmA_F_), 153 °C (SmA_F′_), and 160 °C (N). The substrate surfaces of the cells were coated with PMMA to provide free orientation in the plane. The scale bar in each image represents 100 µm. b) Small‐angle X‐ray diffraction profiles measured at various temperatures during the heating process. c) 2D SAXD images of samples oriented by rubbing in the direction parallel to the equatorial direction. d) Electric displacement–electric field (*D*–*E*) hysteresis curves at 130 °C (SmA_F_), 150 °C (SmA_F′_), and 160 °C (N) with the voltage applied parallel to the director. e) Temperature dependences of dielectric permittivity (green squares) and SHG intensity during application and removal of an electric voltage (red triangles and blue circles, respectively) during the cooling process. Light polarized parallel to the director was incident on the sample for SHG measurements. The low‐temperature phase showed no large dielectric permittivity, probably because the coercive field of this phase was large relative to the electric field applied in the dielectric measurements. In fact, a wide *D*–*E* hysteresis was observed in this phase, as shown in (d) (130 °C).

**Figure** **3** shows the experimental results of **6**. All phase transitions in **6** were enantiotropic, indicating that each phase can be regarded as the most thermodynamically stable phase rather than a metastable supercooled phase. Two smectic phases were observed at the lower‐temperature range of the N phase in **6**. These two smectic phases showed angular mosaic‐like structures as shown in the images at 153 and 135 °C in Figure 3a. Fine streaks were observed in each mosaic of the higher‐temperature phase. The SAXD peaks of both phases appeared at almost the same Bragg angle, 2*θ* = 3.54° (Figures 3b and Figure S5‐2, Supporting Information). The calculated layer spacing (≈2.5 nm) nearly matched the single‐molecule length of **6** (2.25 nm; Section S6, Supporting Information). The WAXD spectra of both phases showed no sharp peaks around 2*θ* = 20°, the region corresponding to the intermolecular lateral distance (Figure S5‐1b, Supporting Information). The small peak at 2*θ* = 7.20° observed at 132.2, 142.0, and 151.1 °C for **6** (Figure S5‐1b, Supporting Information) may be second‐order diffraction of the layer structure since the ratio of the scattering vectors is ≈1:2 for the peak at 2*θ* = 3.54°. Figure 3c shows the 2D SAXD images of **6** sandwiched between rubbed polyimide films. The images of both smectic phases presented clear diffraction spots along the equatorial line parallel to the rubbing direction. The diffraction spots were sharp, especially at the low‐temperature side (135 °C). These phases can be identified as smectic A, considering jointly two observations: 1) a uniform homeotropic orientation can be obtained in cells treated with a surface‐alignment layer to induce a homeotropic orientation (Figure S3‐3, Supporting Information) and 2) uniaxial homogeneous orientation can be obtained in cells with a rubbed polyimide layer (Figure S3‐4, Supporting Information). Notably, at low temperatures, the homeotropic orientation imposed by the surface treatment may not be followed, probably because the sample can deform to relieve the electrostatic repulsion caused by P_S_. In addition, both phases showed *D*–*E* hysteresis along the director direction and SHG activity when light was polarized parallel to the director (Figure 3d,e). Therefore, both phases were identified as ferroelectric smectic A phases exhibiting longitudinal ferroelectricity. Given the similar POM textures of the lower‐temperature phase and the SmA_F_ phase of **4**, the lower‐temperature phase (from melting point to 146 °C) was identified as SmA_F_. The higher‐temperature smectic phase (146–158 °C) was referred to as SmA_F′_ to distinguish it from SmA_F_. The *δɛ*(∥) increased rapidly with decreasing temperature in the SmA_F′_ phase but was innocently smaller in the SmA_F_ phase, probably because the coercive P_S_ field increased in the SmA_F_ phase and was less likely to undergo polarization changes caused by P_S_ from the electric field used in the dielectric measurements. This phase exhibited a wide *D*–*E* hysteresis at 130 °C (Figure 3d). As additional data, Figure S4‐3, Supporting Information, shows the frequency dispersion of the dielectric permittivity at each temperature and Figure S4‐5, Supporting Information, plots the temperature dependence of the dielectric permittivity at different frequencies.

Interestingly, the diffraction spots of the smectic layer in SmA_F_ are streaked in the meridional direction (Figure S5‐3, Supporting Information). If these streaks were due to the orientation distribution of layers, they would be arc shaped, while in Figure S5‐3a, Supporting Information, they clearly extend linearly along the meridian. Therefore, the smectic phase forms anisotropic domains that are extremely small in the direction perpendicular to the director. This phenomenon is especially noticeable in the SmA_F_ and SmX_F_ phases of **4**, suggesting a nematic‐like smectic phase in which elongated smectic domains with a nematic‐like arrangement extend along the director.

**Figure 4:**
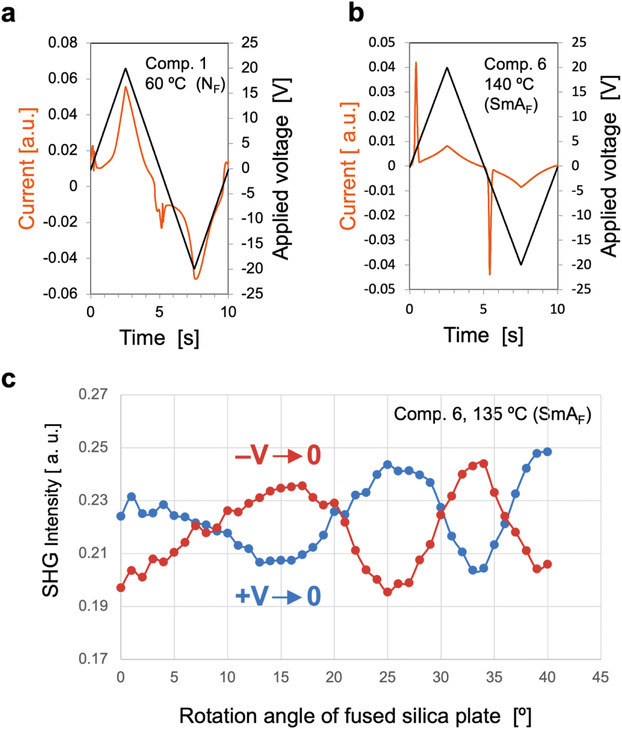
Characterization and self‐retaining properties of longitudinal ferroelectricity. a) Current‐switching response of **1** in a 10‐µm‐thick homeotropic indium tin oxide (ITO) cell under an applied triangular wave (*V*_pp_ = 20 V and *f* = 0.1 Hz) in the N_F_ phase. b) Current‐switching response of **6** in a 10‐µm‐thick homeotropic ITO cell under an applied triangular wave (*V*_pp_ = 20 V and *f* = 0.1 Hz) in the SmA_F_ phase. c) SHG fringe patterns of the SmA_F_ phase in **6** at 135 °C after removing an applied voltage of 80 V between the electrodes with a gap of 500 µm. The horizontal axis of the graph shows the rotation angle of the fused silica plate placed on the optical axis of the laser. When the plate is rotated, the optical phase of the SHG light is shifted from the Y‐cut quartz in front of it and interferes with the SHG light from the sample, causing periodic changes in intensity.

Unlike conventional ferroelectric smectic phases such as the chiral smectic C phase and the smectic phases of bent‐core molecules, which exhibit P_S_ along their molecular lateral faces, the ferroelectric smectic phases considered herein display longitudinal ferroelectricity indicative of the C_∞v_ symmetry. Although N_F_ and SmA_F_ exhibit a common C_∞v_ symmetry at the microscopic level, they should display large differences in their macroscopic physical behaviors, particularly in their memory effects. In the N phase, the elastic constants are extremely small and elastic splay, twist, and bend distortions in the director are easily induced. The occurrence of P_S_ in the N_F_ phase causes orientational distortion that cancels or reduces the P_S_ in the entire system, thereby losing the memory effect for N_F_. Therefore, numerous streaked defects and/or polydomains are generated when the N phase transitions to the N_F_ phase. Although such orientational distortions can be suppressed to some extent by applying an electric field or surface‐anchoring effect, large uniform monodomains are not easily formed in the absence of an external field. Conversely, in the SmA_F_ and SmA_F′_ phases, P_S_‐induced distortions should be inherently suppressed by the relatively robust layer structure. **Figures** **4**a and 4b show the polarization inversion currents in the N_F_ phase of **1** and the SmA_F_ phase of **6**, respectively, measured at low frequency (0.1 Hz). In a previous study, the N_F_ phase of **1** showed a single sharp polarization inversion current at ≈100 Hz.^[^ ^7^ ^]^ However, at frequencies as low as 0.1 Hz, complex currents with multiple separations occurred near zero applied voltage (Figure 4a), suggesting that the macroscopic P_S_ in the N_F_ phase was not maintained at a zero electric field. POM observations showed that the orientation in the N_F_ phase of **1** relaxed under the zero electric field. By contrast, the SmA_F_ phase of **6** showed a clear polarization inversion current even at 0.1 Hz (Figure 4b) and little orientation relaxation under a zero electric field. Further, the POM images confirmed that the macroscopically poled orientation was maintained in the ferroelectric smectic phases. Section S7, Supporting Information, shows the variations in the POM texture when an in‐plane electric field was applied to **6**. In the SmA_F′_ phase at 148 °C, the director remained macroscopically oriented in a single direction even after removing the voltage. Furthermore, the P_S_ of the SmA_F_ phase was maintained after applying and removing the electric field (see the SHGs with the electric field applied and removed in Figures 2e and 3e). In the N_X_ phase, the SHG intensity considerably differed between the on and off states of the electric field (Figure 2e). As shown in Figure S8b of a previous study,^[^ ^7^ ^]^ the SHG intensity of the N_F_ phase (MP phase in ref. [7]) is enhanced under an electric field and relaxes to a lower value after removing the field. This finding indicates that the direction of P_S_ relaxes when the electric field is off in the N_X_ and N_F_ phases. Conversely, the SHG intensity in the SmA_F_ phase shows no substantial difference between the on and off states of the electric field (Figures 2e and 3e). Furthermore, in the SHG interference experiments, the P_S_ in the SmA_F_ phase was clearly inverted after reversing the polarity of the electric field and was preserved after removing the electric field. Figure 4c shows the SHG interference fringe of **6** at 135 °C (SmA_F_ phase) under zero electric field after applying a direct current (DC) electric field. The high/low fringing was reversed after applying and then removing positive and negative DC electric fields. This result indicates that the P_S_ in SmA_F_ was inverted by reversing the polarity of the electric field and the polar orientation was memorized after removing the field. Similar behavior was observed in the SmA_F′_ phase (Section S8, Supporting Information). Materials that allow macroscopic P_S_ in an equilibrium state without relaxation are highly desired for applications in nonlinear optics and energy‐conversion technologies using photovoltaic and other effects.

### 2.4 Relationship between Molecular Structure and Ferroelectric Properties

Clarifying the relationship between molecular structure and ferroelectricity is crucial for designing future novel materials. Compounds **2** and **3** differ from **1** only in the positions of the two fluoride groups on the third phenyl group. Nevertheless, while **1** has a confirmed N_F_ phase, **2** and **3** showed no ferroelectric phase. This difference in the occurrence of the ferroelectric phase can be attributed to the fact that a slight difference in the position of the fluorine substituents induces a relatively large change in the dipole moment along the long axis of the molecule. A recent study showed that the N_F_ phase emerges only after applying a dipole moment of ≥9 D.^[^ ^11^ ^]^ The magnitudes of the dipole moments along the long axes of molecules **1**–**3** were 9.38, 8.13, and 8.31 D, respectively (the bottom part of Table 1). Compound **5** showed no evidence of a ferroelectric phase despite its large dipole moment (>9 D). Although the magnitude of the dipole moment considerably contributes to the molecular origin of the ferroelectric phase, the occurrence of the ferroelectric phase is also sensitive to another factor, that is, intermolecular interactions. Compounds **4** (ferroelectric phase) and **5** (no ferroelectric phase) have different substituents on the third phenyl group: chloride in **5**, fluoride in **6**. Governed by repulsive intermolecular van der Waals forces, the radius of a chloride group is around 1.2 times that of a fluoride group. Meanwhile, governed by the attractive intermolecular interaction due to polarizability, phenyl chloride is approximately five times larger than phenyl fluoride. Therefore, the differences in molecular structure critically influence whether the ferroelectric phase appears and helpful in understanding the molecular origin of the ferroelectric‐phase appearance. Notably, compounds **4** and **6** are slimmer molecules than **1** because the fluoride groups on the third phenyl groups are successively removed, inducing the emergence of ferroelectric smectic phases. Two findings regarding **6** are particularly noteworthy here: 1) **6** exhibits a ferroelectric phase despite a dipole moment of <9 D along the long axis of the molecule and 2) the N phase of **6** directly transitions to the SmA_F′_ and SmA_F_ phases without passing through the N_F_ phase.

### 2.5 Molecular Ordering in the Ferroelectric Smectic A Phase

**Figure 5:**
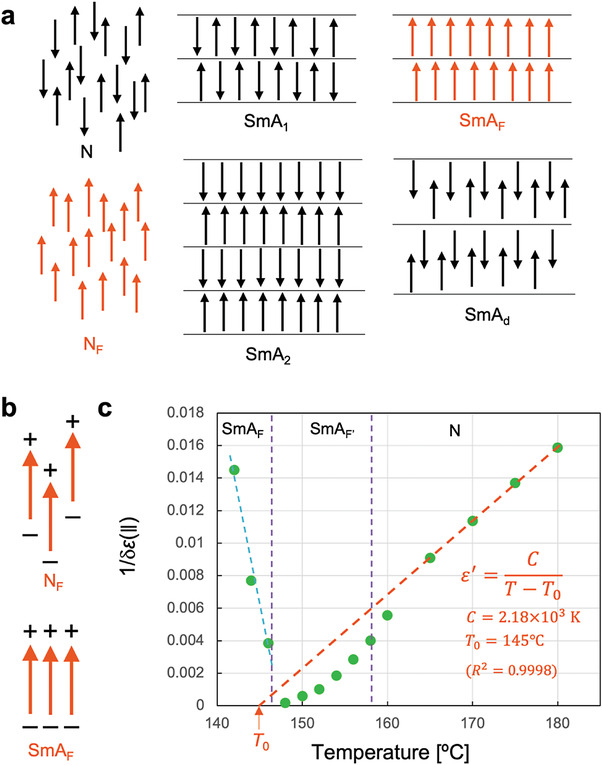
Schematics of various types of molecular alignment in smectic A and Curie–Weiss plot of compound **6**. a) Schematics of layer structure in the normal nematic (N) phase, a ferroelectric nematic (N_F_) phase, a monolayer smectic A phase (SmA_1_), a ferroelectric smectic A phase (SmA_F_), a bilayer smectic A phase (SmA_2_), and a smectic A phase with a periodicity intermediate between monolayer and bilayer (SmA_d_). b) Schematics of charge arrangements in N_F_ and SmA_F_. c) Plot of the reciprocal of the dielectric permittivity versus temperature for **6**; linearity in the plot indicates that the system follows the Curie–Weiss law. The intercept *T*_0_ on the horizontal axis is the Curie–Weiss temperature.

As shown in **Figure** **5**a, the fundamental layer structure of the smectic A phase generally includes SmA_1_ with a layer spacing of approximately a single molecular length, SmA_2_ with a layer spacing equaling two molecules facing each other, and SmA_d_ with a layer spacing determined by partially overlapping molecules. If the smectic A phases of **4** and **6** were SmA_2_ or SmA_d_, a diffraction peak would appear with a spacing of twice the molecular length (i.e., around 2θ = 1.8°) or with a spacing between one and two molecular lengths. However, no peaks appeared at these angles. Because the longest periodic order in the SAXD spectra was equivalent to the length of one molecule along its longitudinal axis (Figures 2b and 3b), the smectic phases observed for **4** and **6** are neither SmA_2_ nor SmA_d_. Their smectic structure is clearly not SmA_1_ with an antiparallel arrangement of dipole moments because the phases exhibit ferroelectricity. From the observed magnitude of P_S_ (3–4 *µ*C cm^−2^), we calculated that the dipole moment in the smectic A phases is oriented almost perfectly along the long axis of the molecule, as observed in the N_F_ phase.^[^ ^7^, ^9^ ^]^ The P_S_ magnitude in these phases is considerably larger than that in conventional ferroelectric smectic phases, about a few 100 nC cm^−2^.^[^ ^15^ ^]^ The SmA_F_ in Figure 5a presents a schematic of the dipole arrangement in the smectic phases of **4** and **6**. We may reasonably assume that if dipole–dipole interactions induce longitudinal ferroelectricity, the arrangement of the same‐polarity parts between laterally adjacent and distant dipoles (as occurs in the N_F_ phase) should be considerably more stable than the arrangement of same‐polarity parts with close lateral distances between dipoles (as occurs in the SmA_F_ phase) (Figure 5b). In this scenario, direct transitions from the N phase to the ferroelectric smectic phase while bypassing the N_F_ phase, as observed in **6**, are unexpected. The dense charge on the layer surface can be efficiently canceled by the opposite‐sign charge of another neighboring layer, reducing the overall electrostatic free energy. This intriguing phenomenon strongly suggests that the layered structure favors the development of longitudinal ferroelectricity or the formation of the layered structure triggers longitudinal ferroelectricity. Fluctuations in the smectic short‐range order or the generation of smectic cybotactic clusters are not unusual in N phases. Smectic short‐range ordering in the N phase might induce the N_F_ phase. Meanwhile, theoretical studies have shown that N_F_ and SmA_F_ can be stabilized when the molecules have a tapered geometry and undergo noncentrosymmetric interactions on the molecular lateral faces.^[^ ^16^ ^]^ Whether such effects influence **6** must be clarified in the future study.

As shown in Figure 5c, a plot of temperature against the reciprocal of the dielectric permittivity leads to an interesting insight. The reciprocal of *δɛ*(∥) in the N phase decreased linearly with temperature and intercepted the temperature axis at *T*_0_ = 145 °C, which is very close to the phase‐transition temperature of SmA_F_ (146 °C). The reciprocal dielectric permittivities at 1 and 10 kHz exhibited similar trends (Figure S9, Supporting Information). From this result, we inferred that the Curie–Weiss law adequately describes the transition from the N phase to the SmA_F_ phase. *T*_0_ corresponds to the Curie–Weiss temperature, and its closeness to the phase‐transition temperature suggests the occurrence of a second‐order or weak first‐order ferroelectric order–disorder transition. Additionally, the observed ferroelectricity is a proper ferroelectricity driven by polarization as the primary order parameter, not an improper ferroelectricity in which the spontaneous polarization is a by‐product of another structural phase transition. If the smectic order induces or enhances the longitudinal ferroelectricity, we could better understand the origin of longitudinal ferroelectricity and provide advance molecular design guidelines for developing such phases.

### 2.6 Why SmA_F_ Phases Lack the Typical Focal Conic (And/or Fan‐Shaped) Textures of Smectic Phases

The presence of P_S_ in the SmA_F_ phase is reflected in the textures observed via POM. The angular mosaic textures of the SmA_F_ phase shown in Figures 2a and 3a present clear difference from the typical textures of smectic A phase textures. In general, focal conic (and/or fan‐shaped) texture is the most common texture observed in a natural smectic A phase. In liquid crystal research, this texture is typically used as the primary indicator to identify the smectic phase. Focal conic textures are formed due to the arrangements of layered tori or so‐called Dupin cyclides, which are feasible when the smectic layers have indistinguishable front and back sides. If the P_S_ occurs along the layer normal in the layered tori and Dupin cyclide structures, the polarity of the spontaneous polarization ***P*** will be focused (div ***P*** < 0) or divergent (div ***P*** > 0) and opposed on the centerlines of the tube and torus (see Section S10, Supporting Information). This arrangement is electrostatically disadvantageous. Therefore, tori and Dupin cyclide structures are forbidden in the SmA_F_ phase and the layers are mainly arranged in parallel. Hence, angular mosaic textures are preferentially formed and focal conic and fan‐shaped textures are not observed.

## 3 Conclusion

Novel ferroelectric smectic A phases were developed by simplifying the molecular structure of compound **1**, which is known to exhibit the N_F_ phase. The magnitude of P_S_ induced in the ferroelectric smectic A phase suggests a nearly complete polar ordering. The macroscopic C_∞v_ symmetry in the discovered ferroelectric phases is the highest symmetry found thus far in layered ferroelectrics. Compound **6** undergoes a direct transition from the N phase to the ferroelectric smectic phase, bypassing the N_F_ phase, with a dipole moment of <9 D. This result suggests that the ferroelectric smectic A phase is generated via a mechanism other than the secondary effect of N_F_ phase development. The N‐to‐SmA_F_ phase transition obeyed the Curie–Weiss law, which is valid in principle for ferroelectric order–disorder transitions. The smectic phase develops proper longitudinal ferroelectricity, in which electrostatically unfavorable alignment of the dipole moments should occur in the plane. This phenomenon opens new avenues into ferroelectric soft matter. In the ferroelectric smectic A phase, the macroscopic orientation of P_S_ is maintained even after the removal of the electric field. This persistence is extremely beneficial in practical applications of the ferroelectric smectic A phase. In general, increasing the symmetry and fluidity of a material weakens its ferroelectricity and high fluidity reduces its memory ability. The ferroelectric smectic phases found in this study uniquely possess high symmetry, fluidity, and memory ability.

## 4 Experimental Section

All compounds used in this study could chemically transform or degrade at temperatures above 120 °C. Therefore, measurements at high temperatures were performed as quickly as possible.

### Molecular Syntheses

The synthetic reaction schemes and molecular characterizations of compounds **2**–**6** are described in Section S1, Supporting Information.

### Differential Scanning Calorimetry

DSC measurements were performed using a DSC 1 STAR^e^ System calorimeter (Mettler Toledo, Switzerland) with a dedicated aluminum pan at a scanning rate of 5 °C min^−1^.

### Polarized Optical Microscopy

POM was conducted using Nikon ECLIPSE LV100NPOL with a DS‐Ri2 camera using four types of cells: no orientation treatment, homogeneous orientation (parallel to the substrate in one direction), planar orientation (parallel to the substrate but with free in‐plane orientation), and homeotropic orientation (perpendicular to the substrate). The homogeneous‐orientation cells were polyimide‐rubbed cells manufactured by E.H.C., and the planar orientation cells were developed using poly(methyl methacrylate) (PMMA)‐coated glass substrates. Homeotropic orientation was achieved via spin coating and drying a cyclopentanone solution of octadecyltrimethoxysilane on a glass substrate, followed by heat treatment at 130 °C for 3 h under vacuum to chemically modify the substrate surface. After two iterations of this process, substrates with vertical orientation were produced.

### Small‐Angle X‐Ray Diffraction

SAXD was performed using a Bruker NANOSTAR system with a Cu K*α* radiation source (50 kV and 100 mA) and a camera length of 276 mm. The heating stage was an MRI TCPU H, and the detector was a Vantec2000. The samples were melted and poured into the hole of the sample holder (diameter = 4 mm and depth = 1 mm). The bottom faces of the hole were covered with Kapton tape. For orientation measurements, the cells with 25 µm‐thickness sandwiched between two anti‐parallel rubbed Kapton tapes were used.

### Wide‐Angle X‐Ray Diffraction

WAXD was performed using Rigaku SmartLab with a CuKαa radiation source (45 kV and 200 mA) and a PILATUS (**4**) and SC‐70 (**6**) detector. The samples were mounted on an Al pan for simultaneous WAXD and DSC measurements.

### Dielectric Permittivity

The dielectric relaxation spectra were measured by acquiring the magnitude and phase of the complex impedance in the range of 1 Hz–10 MHz using an impedance/gain‐phase analyzer (SI 1260, Solatron Metrology). The applied voltage was set to 0.1 V_rms_. The homeotropically oriented cells (electrode area = 1 cm^2^ and cell thickness = 10 µm) were filled with the liquid crystal samples for measurements. The indium tin oxide (ITO) electrode resistance and capacitance were measured in advance using an empty cell, and these values were subtracted from the impedance of each sample to calculate the dielectric permittivity. The *ɛ*′(∥) values presented in Table 1 were measured in a cell fabricated from substrates with transparent ITO electrodes subjected to vertically oriented surface treatment. The dielectric relaxation strength *δɛ*(∥) was obtained from the Cole–Cole plot, which plots *ɛ*″(∥) versus *ɛ*′(∥) as a function of frequency.

### Second Harmonic Generation

During the SHG measurements, a Q‐switched ND:YAG laser (LS‐2130, LOTIS TII; wavelength = 1064 nm, energy = 75 µJ, pulse width = 10 ns, and pulse period = 20 Hz) was incident on homeotropically oriented cells (electrode area = 1 cm^2^ and cell thickness = 10 µm) or planar oriented cells (PMMA coating) filled with liquid crystal samples. The SHG interferometry was performed as described in a previous paper^[^ ^7^ ^]^ and its Supporting Information. Planar orientation cells were used as samples.

### Polarization Current and *D*–*E* Hysteresis

For polarization reversal current measurements, a waveform generator (2411B, Toyo Technica), an analog‐to‐digital converter (WaveBook 516A, Toyo Technica), and a current–voltage/charge–voltage (I–V/Q–V) converter (Model 6254C, Toyo Technica) were constructed. The sample was injected into a homeotropically oriented ITO cell (electrode area = 1 cm^2^ and cell thickness = 10 µm) coated with a silane coupling agent, and the polarization reversal current was measured using the triangular wave method. The *D*–*E* hysteresis loops were obtained by integrating the measured currents. The voltage and frequency ranges of the measurements were −20–+20 V and 0.1–100 Hz, respectively.

### Calculation of Dipole Moment

Structural optimization of **1**–**6** and the magnitudes of the dipole moment *µ* and angle *θ* of the dipole moment with respect to the long axis of the molecules were calculated using density functional theory with the B3LYP/6‐31+G(2d,p) functional implemented by the Gaussian09 program package.^[^ ^17^ ^]^

## Conflict of Interest

The authors declare no conflict of interest.

## Author Contributions

H.K. supervised the project and wrote the manuscript. H.M. performed chemical synthetic experiments and chemical analyses. K.I. and S.E. performed measurements and data analyses regarding phase‐transition behavior (POM and DSC), dielectric and current properties, and SHG properties. S.A. performed XRD measurements and data analyses. Y.O. prepared the setup of the apparatus and measurement programs. All authors discussed the results and contributed to the manuscript.

## Supporting information

Supporting Information

## Data Availability

The data that support the findings of this study are available from the corresponding author upon reasonable request.
