# Supplementary material for: Fluid Layered Ferroelectrics with Global C∞v Symmetry
Source: Adv Sci (Weinh). 2022 Jul 22;9(26):2202048. doi: 10.1002/advs.202202048 (PMC9475520; doi:10.1002/advs.202202048)
Supplement: Supplementary file 1 — Supporting Information [file ADVS-9-2202048-s001.pdf]

Supporting Information

Fluid Layered Ferroelectrics with Global $C_{\infty v}$ Symmetry

Hirotsugu Kikuchi[1], Hiroyuki Matsukizono[1], Koki Iwamatsu[2], Sota Endo[2], Shizuka Anan[1], Yasushi Okumura[1]

[1]Kyushu University, Institute for Materials Chemistry and Engineering
[2]Kyushu University, Interdisciplinary Graduate School of Engineering Sciences

SI-1. The synthetic reaction schemes and molecular characterization of the compounds 2-6

Reagents

Anhydrous magnesium sulfate ($MgSO_4$), anhydrous sodium sulfate (Na_2SO_4), p-toluenesulfonate monohydrate ($TsOH{\cdot}H_2O$) and 2-n-propyl-1,3-propanediol were obtained from FUJIFILM Wako Pure Chemical Co., Ltd. n-butyl lithium hexane solution (1.6 mol L^{-1}), 1-ethyl-3-(3-diaminopropyl)carbodiimide hydrochloride (EDC·HCl = WSC), 4-dimethylaminopyridine (DMAP), 4-bromo-3-chlorobenzaldehyde, methyl terephthalaldehydate and other fluorinated benzene derivatives were purchased from Tokyo Chemical Industry Co., Ltd. 4-(3,4,5-trifluorophenyl)-3-fluorophenol, was synthesized according to literature procedure or obtained from JNC Co., Ltd. These reagents and solvents were used without any purification.

Measurements

NMR spectra were recorded on a JNM-ECZ400 spectrometer (JEOL Co., Ltd.) at 400 MHz for ^{1}H NMR, 100 MHz for ^{13}C NMR and 376 MHz for ^{19}F NMR. Chemical shifts in ^{1}H and ^{13}C NMR spectra are given in parts per million (ppm) downfield using tetramethylsilane as an internal standard. For ^{19}F NMR, trifluoroacetic acid was used. The high resolution mass spectroscopies (HRMS) were conducted on a JMS-700 instrument (JEOL Co., Ltd.) with an electron ionization mode.

Synthesis

1-1. Synthesis of 3-fluoro-4-(3,4,5-trifluorophenyl)phenyl 2,5-difluoro-4-(trans-5-n-propyl-1,3-dioxan-2-yl)benzoate (2)

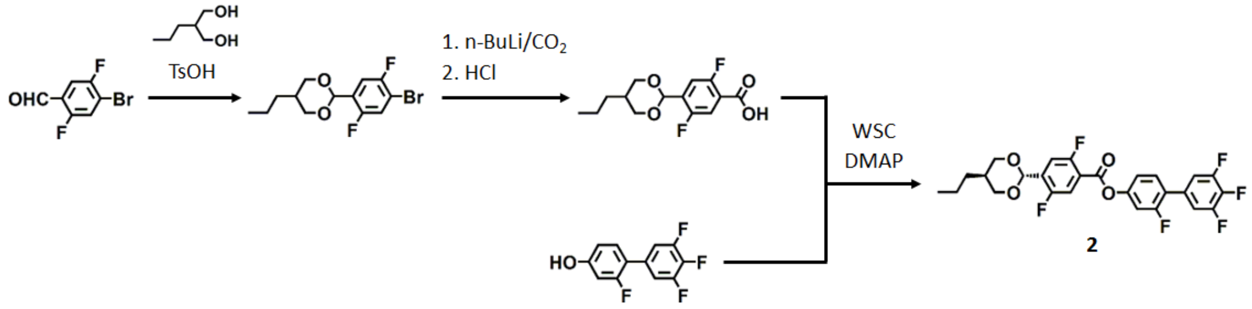

1-1-1. 1-bromo-2,5-Difluoro-4-(5-n-propyl-1,3-dioxan-2-yl)benzene

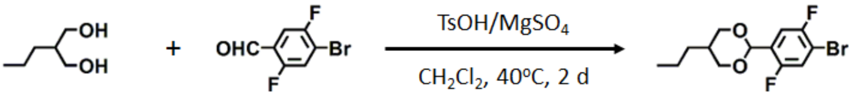

To a CH_2Cl_2 (30 mL) of 4-bromo-2,5-difluorobenzaldehyde 4.90 g (22.2 mmol), 2-n-propyl-1,3-propanediol 2.94 g (24.9 mmol) were added TsOH· H_2O 454 mg (2.39 mmol) and anhydrous $MgSO_4$ 14.0 g (117 mmol). After stirred at 40°C for 2 days, the mixture was poured into cold water (200 mL) containing $NaHCO_3$ 2.0 g (24 mmol). Oily fractions were extracted with CH_2Cl_2 (200 mL) and the organic layer was washed with distilled water (200 mL) and then dried over anhydrous Na_2SO_4. After evaporation, the residues were allowed to stand overnight. The resulting solids were collected and washed with n-hexane to give white solids in 95% trans-form. Yield: 887 mg (12.4%). ^{1}H NMR (400 MHz, DMSO-d_6, ppm): δ 7.77 (dd, *J* = 9.2, 5.6 Hz, 1H; phenyl), 7.42 (dd, *J* = 8.8, 12 Hz, 1H; phenyl), 5.72 (s, 0.05H; cis-form acetal), 5.65 (s, 0.95H; trans-form acetal), 4.13 (dd, *J* = 12, 4.0 Hz, 1.9H; -CH_2-O- in trans-form), 4.07-3.93 (m, 0.2H; cis-form -CH_2-O-), 3.55 (t, *J* = 12 Hz, 1.9H; -CH_2-O- in trans-form), 2.04-1.93 (m, 0.95H; -C<u>H</u>-CH_2O- in trans-form), 1.71-1.65 (m, 0.1H; CH_3-CH_2-C<u>H</u>$_2$- in cis-form), 1.44-1.23 (m, 2H; -C<u>H</u>-CH_2O- in cis-form, CH_3-C<u>H</u>$_2$-CH_2-), 1.04 (q, *J* = 7.5 Hz, 1.9H; CH_3-CH_2-C<u>H</u>$_2$- in trans-form), 0.94-0.85 (m, 3H; CH_3-).

1-1-2. 2,5-Difluoro-4-(5-n-propyl-1,3-dioxan-2-yl)benzoic acid

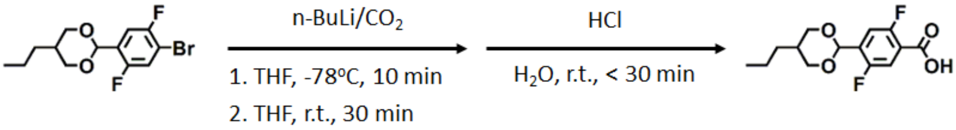

To a THF solution (12 mL) of 1-bromo-2,5-difluoro-4-(5-n-propyl-1,3-dioxan-2-yl)benzene 1.74 g (5.42 mmol) was added 1.6 M n-butyl lithium n-hexane solution (4.0 mL, 6.4 mmol, 1.2 equiv.) at -78°C using a bath of dry ice/acetone. After stirring for several minutes, dry ice 7 g was added in the solution and then the mixture was stirred at -78°C for < 10 min and then stirred at r.t. for 30 min. After quenching using H_2O (1 mL), the mixture was concentrated and then added in distilled water (100 mL). After washed with CH_2Cl_2 (100 mL), the aqueous layer was collected and then its pH was adjusted to ca. 3. The resulting solids were collected by suction filtration and washed with distilled water. After drying, white solids were obtained with a cis/trans ratio of 1:4. Yield: 707 mg (45.7%). ^{1}H NMR (400 MHz, $CDCl_3$, ppm): δ 7.77 (dd, *J* = 9.2, 5.6 Hz, 1H; phenyl), 7.47 (dd, *J* = 11, 6.0 Hz, 1H; phenyl), 5.72 (s, 0.2H; cis-form acetal), 5.68 (s, 0.8H; trans-form acetal), 4.23 (dd, *J* = 12, 4.8 Hz, 1.9H; -CH_2-O- in trans-form), 4.14-4.06 (m, 0.2H; cis-form -CH_2-O-), 3.56 (t, *J* = 12 Hz, 1.9H; -CH_2-O- in trans-form), 2.20-2.15 (m, 0.9H; -C<u>H</u>-CH_2O- in trans-form), 1.83-1.77 (m, 0.1H; CH_3-CH_2-C<u>H</u>$_2$- in cis-form), 1.47-1.29 (m, 2H; -C<u>H</u>-CH_2O- in cis-form, CH_3-C<u>H</u>$_2$-CH_2-), 1.10 (q, *J* = 7.5 Hz, 1.9H; CH_3-CH_2-C<u>H</u>$_2$- in trans-form), 1.00-0.92 (m, 3H; CH_3-).

1-1-3. 3-Fluoro-4-(3,4,5-trifluorophenyl)phenyl 2,5-difluoro-4-(trans-5-n-propyl-1,3-dioxan-2-yl)benzoate (**2**)

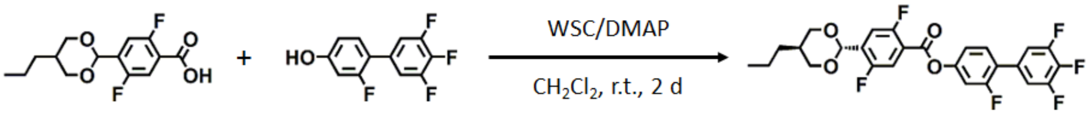

2

To CH_2Cl_2 (7 mL), 2,5-difluoro-4-(5-n-propyl-1,3-dioxan-2-yl)benzoic acid 688 mg (2.4 mmol) and WSC 572 mg

(2.98 mmol) were added and the solution was stirred at ambient temperature for 40 min. To the solution, 4-(3,4,5-trifluorophenyl)-3-fluorophenol 700 mg (2.89 mmol) and DMAP 15 mg (0.12 mmol) were added and the solution was further stirred at rt. for 2 days. The solution was added in CH_2Cl_2 (50 mL) and washed with distilled water (50 mL x 3). The organic layer was dried with anhydrous Na_2SO_4 and then evaporated. The resulting solids were purified by reprecipitation from CH_2Cl_2 MeOH and recrystallization from CH_2Cl_2/MeOH to give white solids in trans-form. Yield: 464 mg (37.9%). ^{1}H NMR (400 MHz, $CDCl_3$, ppm): δ 7.77 (dd, J = 9.2, 5.2 Hz, 1H; phenyl), 7.53 (dd, J = 11, 4.8 Hz, 1H; phenyl), 7.44 (t, J = 6.8 Hz, 1H; phenyl), 7.20-7.12 (m, 4H; phenyl), 5.71 (s, 1H; acetal), 4.25 (dd, J = 12, 4.6 Hz, 2H; $-CH_2-O-$), 3.58 (t, J = 12 Hz, 2H; $-CH_2-O-$), 2.22-2.14 (m, 1H; $-C\underline{H}-CH_2O-$), 1.40-1.31 (m, 2H; $CH_3-C\underline{H_2}-CH_2-$), 1.14-1.08 (m, 2H; $CH_3-CH_2-C\underline{H_2}-$), 0.94 (t, J = 7.8 Hz, 3H; CH_3-). $^{13}C\{^1H\}$ NMR (100 MHz, $CDCl_3$, ppm): δ 161.0 (s, C=O), 159.3 (d, $^1J_{C-F}$ = 249 Hz, C-F in phenol), 158.3 (d, $^1J_{C-F}$ = 258 Hz, C-F in benzoate), 155.3 (d, $^1J_{C-F}$ = 246 Hz, C-F in benzoate), 151.2 (ddd, $^1J_{C-F}$ = 248 Hz, $^2J_{C-F}$ = 10 Hz, $^3J_{C-F}$ = 4 Hz, C-F in trifluorophenyl), 151.0 (d, $^3J_{C-F}$ = 11 Hz, C-O in phenol), 139.5 (dt, $^1J_{C-F}$ = 251 Hz, $^2J_{C-F}$ = 15 Hz, C-F in trifluorophenyl), 133.3 (dd, $^2J_{C-F}$ = 14 Hz, $^3J_{C-F}$ = 8 Hz, benzoate), 130.8 (dt, $^3J_{C-F}$ = 12 Hz, $^4J_{C-F}$ = 3 Hz, trifluorophenyl), 130.6 (d, $^3J_{C-F}$ = 4 Hz, phenol), 124.2 (d, $^2J_{C-F}$ = 12 Hz, phenol), 118.7 (d, $^2J_{C-F}$ = 26 Hz, benzoate), 118.5 (dd, $^2J_{C-F}$ = 12 Hz, $^3J_{C-F}$ = 8 Hz, benzoate), 118.1 (d, $^4J_{C-F}$ = 3 Hz, phenol), 116.9 (dd, $^2J_{C-F}$ = 26 Hz, $^3J_{C-F}$ = 4 Hz, benzoate), 113.2 (ddd, $^2J_{C-F}$ = 15 Hz, $^3J_{C-F}$ = 7 Hz, $^4J_{C-F}$ = 3 Hz, trifluorophenyl), 110.6 (d, $^2J_{C-F}$ = 26 Hz, phenol), 94.9 (d, $^3J_{C-F}$ = 3 Hz, acetal), 72.7 (s, C-O in dioxane), 33.9 (s, $CH_3-CH_2-CH_2-\underline{C}$), 30.2 (s, $CH_3-CH_2-\underline{C}H_2$), 19.5 (s, $CH_3-\underline{C}H_2$), 14.2 (s, CH_3). ^{19}F NMR (376 MHz, $CDCl_3$, ppm): d -113.5 (1H, benzoate), -115.5 (1H, phenol), -124.7 (2H, trifluorophenyl), -162.4 (1H, trifluorophenyl). HRMS (EI) m/z: [M^+] calcd for $C_{26}H_{20}F_6O_4$: 510.1266; found: 510.1267. Anal. calcd for $C_{26}H_{20}F_6O_4$: C 61.18, H 3.95; found: C 61.16, H 3.78.

1-2. Synthesis of 3-fluoro-4-(3,4,5-trifluorophenyl)phenyl 2,3-difluoro-4-(trans-5-n-propyl-1,3-dioxan-2-yl)benzoate (3)

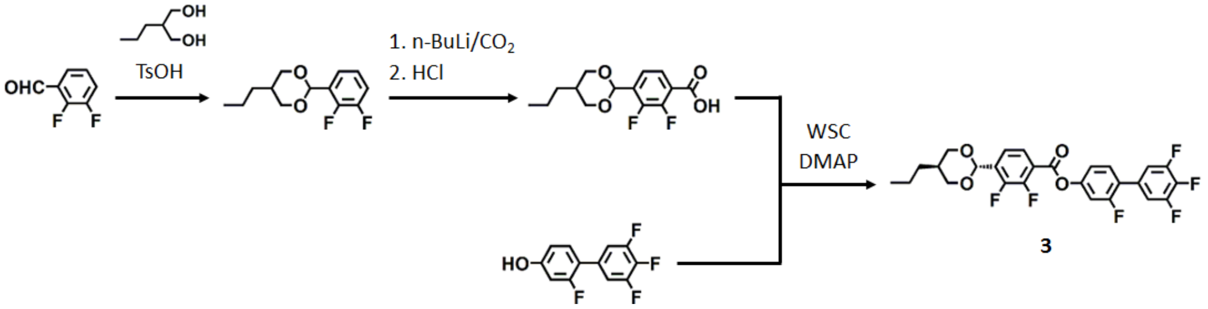

1-2-1. 2,3-Difluoro-1-(5-n-propyl-1,3-dioxan-2-yl)benzene

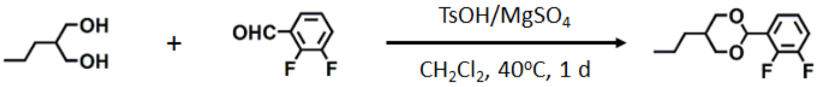

To CH_2Cl_2 (40 mL) were added 2,3-difluorobenzaldehyde 5.00 g (35.2 mmol), 2-n-propl-1,3-propanediol 4.57 g (38.7 mmol), TsOH· H_2O 666 mg (3.50 mmol) and anhydrous $MgSO_4$ 14.5 g (120 mmol). The mixture was stirred at 40°C overnight and then poured into cold water (250 mL) containing $NaHCO_3$ 1.0 g (120 mmol). Oily fractions were extracted with CH_2Cl_2 (250 mL) and the organic layer was washed with distilled water (250 mL x 3) and then dried over anhydrous Na_2SO_4. After evaporation and dryness under reduced pressure, colorless

liquids were obtained with a cis/trans ratio of 2/3. Yield: 8.77 g (>99%). ^{1}H NMR (400 MHz, DMSO-d_6, ppm): δ 7.48-7.41 (m, 1H; phenyl), 7.37-7.33 (m, 1H; phenyl), 7.25-7.20 (m, 1H; phenyl), 5.78 (s, 0.4H; cis-form acetal), 5.71 (s, 0.6H; trans-form acetal), 4.14 (dd, *J* = 12, 4.8 Hz, 1.3H; -CH_2-O- in trans-form), 4.11-3.94 (m, 1.4H; cis-form -CH_2-O-), 3.55 (t, *J* = 12 Hz, 1.3H; -CH_2-O- in trans-form), 2.04-1.95 (m, 0.6H; -C<u>H</u>-CH_2O- in trans-form), 1.70 (q, *J* = 7.5 Hz, 0.7H; CH_3-CH_2-C<u>H</u>$_2$- in cis-form), 1.46-1.24 (m, 2.4H; -C<u>H</u>-CH_2O- in cis-form, CH_3-C<u>H</u>$_2$-CH_2-), 1.02 (q, *J* = 7.5 Hz, 1.2H; CH_3-CH_2-C<u>H</u>$_2$- in trans-form), 0.94-0.86 (m, 3H; CH_3-).

1-2-2. 2,3-Difluoro-4-(5-n-propyl-1,3-dioxan-2-yl)benzoic acid

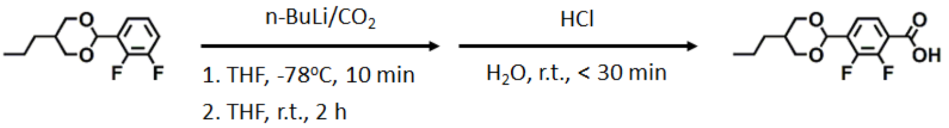

To a THF solution (80 mL) of 2,3-difluoro-1-(5-n-propyl-1,3-dioxan-2-yl)benzene 8.77 g (35.2 mmol) was added dropwise 1.6 M n-butyl lithium n-hexane solution (27 mL, 43.2 mmol) at -78°C using a dry ice/acetone bath. After stirring at -78°C for 10 min, dry ice 30 g was added and then mixture was stirred at the temperature for 10 min. water (1 mL) was added in the mixture and then solvents were evaporated. After washing with CH_2Cl_2 (250 mL)/H_2O (250 mL), the aqueous layer was collected and then its pH was adjusted to ca. 3 by an addition of HCl aq. The resulting precipitates were collected by suction filtration and then washed with distilled water. After drying under reduced pressure, white solids were obtained with a cis/trans ratio of 2:3. Yield: 9.06 g (89.9%). ^{1}H NMR (400 MHz, DMSO-d_6, ppm): δ 13.6 (br, 1H; COOH), 7.72-7.67 (m, 1H; phenyl), 7.44-7.41 (m, 1H; phenyl), 5.82 (s, 0.4H; cis-form acetal), 5.74 (s, 0.6H; trans-form acetal), 4.17-3.95 (m, 2.7H; -CH_2-O-), 3.58 (t, *J* = 11 Hz, 1.3H; -CH_2-O- in trans-form), 2.05-1.96 (m, 0.6H; -C<u>H</u>-CH_2O- in trans-form), 169 (q, *J* = 7.9 Hz, 0.7H; CH_3-CH_2-C<u>H</u>$_2$- in cis-form), 1.46-1.24 (m, 2.4H; -C<u>H</u>-CH_2O- in cis-form, CH_3-C<u>H</u>$_2$-CH_2-), 1.05 (q, *J* = 7.6 Hz, 1.2H; CH_3-CH_2-C<u>H</u>$_2$- in trans-form), 0.94-0.85 (m, 3H; CH_3-).

1-2-3. 3-Fluoro-4-(3,4,5-Trifluorophenyl)phenyl 2,3-difluoro-4-(trans-5-n-propyl-1,3-dioxan-2-yl)benzoate (**3**)

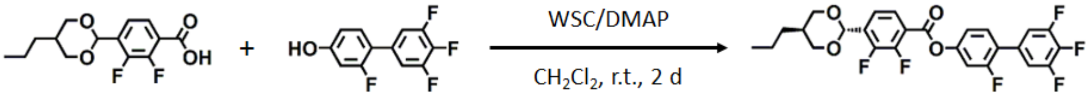

3

To a CH_2Cl_2 solution (20 mL) of 2,3-difluoro-4-(5-n-propyl-1,3-dioxan-2-yl)benzoic acid 2.86 g (10.0 mmol) was added WSC 2.30 g (12.0 mmol). The solution was stirred at ambient temperature for 30 min, 3-fluoro-4-(3,4,5-trifluorophenyl)phenol 2.91 g (12.0 mmol) and DMAP 61 mg (0.50 mmol) were added. After the solution was stirred at r.t. for 2 days, the solution was added in CH_2Cl_2 (150 mL) and then washed with distilled water (150 mL x 3). The organic layer was dried over anhydrous Na_2SO_4 and then concentrated. After reprecipitation from CH_2Cl_2/MeOH, the solids were purified by silica-gel column chromatography (n-hexane/EtOAc volume ratio of 1:3) to give white solids in trans-form. Yield: 1.04 g (24.3%). ^{1}H NMR (400 MHz, $CDCl_3$, ppm): δ 7.86 (td, *J* = 7.2, 2.0 Hz, 1H; phenyl), 7.54 (t, *J* = 6.8 Hz, 1H; phenyl), 7.44 (t, *J* = 8.8 Hz, 1H; phenyl), 7.20-7.13 (m; 4H; phenyl), 5.75 (s, 1H; acetal), 4.25 (dd, *J* = 12, 4.8 Hz, 2H; -CH_2-O-), 3.59 (t, *J* = 12 Hz, 2H; -CH_2-O-), 2.24-2.15 (m, 1H; -C<u>H</u>-CH_2O-), 1.40-1.31 (m, 2H; CH_3-C<u>H</u>$_2$-CH_2-), 1.11 (q, *J* = 7.7 Hz, 2H; CH_3-CH_2-C<u>H</u>$_2$-), 0.94 (t, *J* = 7.8 Hz, 3H; CH_3-). ^{13}C{^{1}H} NMR (100 MHz, $CDCl_3$, ppm): δ 161.2 (d, $^{3}J_{C\text{-}F}$ = 3 Hz, C=O), 159.3 (d, $^{1}J_{C\text{-}F}$ = 249 Hz, C-F in

phenol), 151.2 (ddd, ${}^1J_{C\text{-}F}$ = 249 Hz, ${}^2J_{C\text{-}F}$ = 11 Hz, ${}^3J_{C\text{-}F}$ = 4 Hz, C-F in trifluorophenyl), 151.0 (d, ${}^3J_{C\text{-}F}$ = 12 Hz, C-O in phenol), 150.6 (dd, ${}^1J_{C\text{-}F}$ = 264 Hz, ${}^2J_{C\text{-}F}$ = 14 Hz C-F in benzoate), 148.9 (dd, ${}^1J_{C\text{-}F}$ = 252 Hz, ${}^2J_{C\text{-}F}$ = 13 Hz, C-F in benzoate), 139.5 (dt, ${}^1J_{C\text{-}F}$ = 252 Hz, ${}^2J_{C\text{-}F}$ = 15 Hz, C-F in trifluorophenyl), 133.3 (d, ${}^2J_{C\text{-}F}$ = 10 Hz, benzoate), 130.8 (dt, ${}^3J_{C\text{-}F}$ = 12 Hz, ${}^4J_{C\text{-}F}$ = 3 Hz, trifluorophenyl), 130.6 (d, ${}^3J_{C\text{-}F}$ = 4 Hz, phenol), 126.5 (d, ${}^3J_{C\text{-}F}$ = 4 Hz, benzoate), 124.2 (d, ${}^2J_{C\text{-}F}$ = 12 Hz, phenol), 122.0 (s, benzoate), 119.2 (d, ${}^2J_{C\text{-}F}$ = 7 Hz, benzoate), 118.1 (d, ${}^4J_{C\text{-}F}$ = 4 Hz, phenol), 113.2 (ddd, ${}^2J_{C\text{-}F}$ = 15 Hz, ${}^3J_{C\text{-}F}$ = 6 Hz, ${}^4J_{C\text{-}F}$ = 3 Hz, trifluorophenyl), 110.6 (d, ${}^2J_{C\text{-}F}$ = 26 Hz, phenol), 95.3 (d, ${}^3J_{C\text{-}F}$ = 4 Hz, acetal), 72.8 (s, C-O in dioxane), 33.9 (s, CH_3-CH_2-CH_2-C̲), 30.2 (s, CH_3-CH_2-C̲H_2), 19.5 (s, CH_3-C̲H_2), 14.2 (s, CH_3). ^{19}F NMR (376 MHz, $CDCl_3$, ppm): d -115.6 (1H, phenol), -134.5 (1H, benzoate), -135.5 (2H, trifluorophenyl), -143.1 (1H, benzoate), -162.5 (1H, trifluorophenyl). HRMS (EI) *m/z*: [M^+] calcd for $C_{26}H_{20}F_6O_4$: 510.1266; found: 510.1267. Anal. calcd for $C_{26}H_{20}F_6O_4$: C 61.18, H 3.95; found: C 61.34, H 3.81.

1-3. Synthesis of 3-fluoro-4-(3,4,5-trifluorophenyl)phenyl 2-fluoro-4-(trans-5-n-propyl-1,3-dioxan-2-yl)benzoate (4)

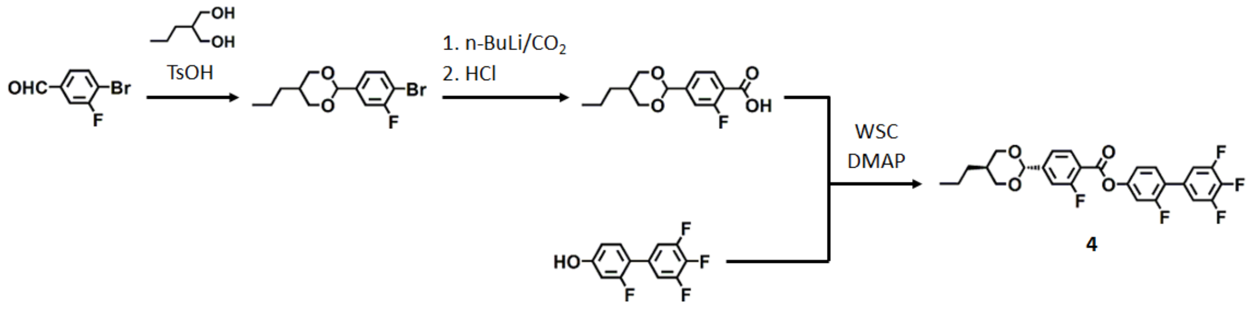

1-3-1. 4-Bromo-3-fluoro-1-(5-n-propyl-1,3-dioxan-2-yl)benzene

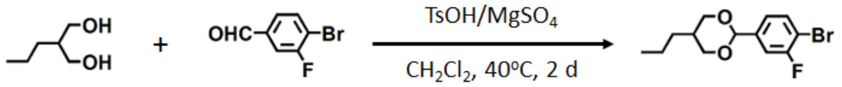

To CH_2Cl_2 (30 mL) were added 2-n-propyl-1,3-propanediol 3.40 g (29.0 mmol) and 4-bromo-3-fluorobenzaldehyde 4.87 g (24.0 mmol). To the solution, TsOH H_2O 470 mg (2.47 mmol) and anhydrous $MgSO_4$ 24.0 g (200 mmol) were added and then the mixture was heated at 40°C for 2 days. After cooled to ambient temperature, the mixture was poured into cold water (100 mL). After extraction with CH_2Cl_2 (200 mL), the organic layer was washed twice with distilled water (200 mL) and then dried over anhydrous Na_2SO_4. The organic layer was evaporated and dried under reduced pressure to give colorless liquids in a quantitative yield. The cis/trans ratio was 1:4. Yield: 7.33 g. ^{1}H NMR (400 MHz, DMSO-d_6, ppm): δ 7.01 (t, *J* = 7.4 Hz, 1H; phenyl), 7.33 (d, *J* = 10 Hz, 1H; phenyl), 7.21 (d, *J* = 8.0 Hz, 1H; phenyl), 5.53 (s, 0.2H; cis-form acetal), 5.46 (s, 0.8H; trans-form acetal), 4.13 (d, *J* = 10 Hz, 1.5H; -CH_2-O- in trans-form), 4.07-3.93 (m, 1H; cis-form -CH_2-O-), 3.51 (t, *J* = 10 Hz, 1.5H; -CH_2-O- in trans-form), 1.97-1.95 (m, 0.8H; -CH̲-CH_2O- in trans-form), 1.67-1.62 (m, 0.5H; CH_3-CH_2-CH̲$_2$- in cis-form), 1.44 (m, 0.2H; -CH̲-CH_2O- in cis-form), 1.39-1.33 (m, 0.5H, CH_3-CH̲$_2$-CH_2- in cis-form), 1.31-1.25 (m, 1.5H; CH_3-CH_2-CH̲$_2$- in trans-form), 1.06-1.03 (m, 1.5H, CH_3-CH̲$_2$-CH_2- in trans-form), 0.93-0.86 (m, 3H; CH_3-).

1-3-2. 2-Fluoro-4-(5-n-propyl-1,3-dioxan-2-yl)benzoic acid

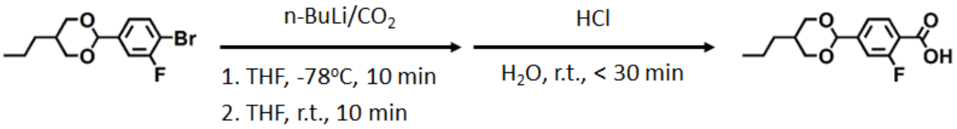

To dehydrated THF (20 mL) was added 4-bromo-3-fluoro-1-(5-n-propyl-1,3-dioxane-2-yl)benzene 3.95 g (13.0 mmol) and then the solution was cooled to -78°C using a bath of CO_2/acetone. To the solution, 1.6 M n-butyl lithium hexane solution (10 mL, 16 mmol, 1.2 equiv.) was slowly added and then stirred at -78°C for 10 min. After that, dry ice 20 g (455 mmol) was added and further stirred at -78°C for 10 min and then stirred at rt. for 30 min. To the mixture, water (1 mL) was added and then evaporated to remove solvents. The residues were dissolved in distilled water (200 mL) and the pH of the solution was adjusted to 2-3 by an addition of 1 M HCl aqueous solution. The resulting precipitate was collected by suction filtration and washed thoroughly with distilled water. After drying under reduced pressure, white solids were obtained with a cis/trans ratio of 3:17. Yield: 2.33 g (66.7%). ^{1}H NMR (400 MHz, DMSO-d_6, ppm): δ 13.3 (br, 1H; CCOH), 7.87 (t, *J* = 8.2 Hz, 1H; phenyl), 7.27 (d, *J* = 12 Hz, 1H; phenyl), 7.21 (d, *J* = 8.0 Hz, 1H; phenyl), 5.58 (s, 0.15H; cis-form acetal), 5.51 (s, 0.85H; trans-form acetal), 4.50 (dd, *J* = 12, 5.0 Hz, 1.7H; -CH_2-O- in trans-form), 4.09-3.94 (m, 1H; cis-form -CH_2-O-), 3.53 (t, *J* = 12 Hz, 1.7H; -CH_2-O- in trans-form), 2.02-1.93 (m, 0.85H; -C$\underline{H}$-CH_2O- in trans-form), 1.65 (q, *J* = 7.9 Hz, 0.3H; CH_3-CH_2-C$\underline{H}_2$- in cis-form), 1.45 (m, 0.15H; -C$\underline{H}$-CH_2O- in cis-form), 1.39-1.34 (m, 0.3H, CH_3-C$\underline{H}_2$-CH_2- in cis-form), 1.31-1.24 (m, 1.7H; CH_3-CH_2-C$\underline{H}_2$- in trans-form), 1.04 (q, *J* = 7.4 Hz, 1.7H, CH_3-C$\underline{H}_2$-CH_2- in trans-form), 0.93-0.85 (m, 3H; CH_3-).

1-3-3. 3-Fluoro-4-(3,4,5-trifluorophenyl)phenyl 2-fluoro-4-(trans-5-n-propyl-1,3-dioxan-2-yl)benzoate (**4**)

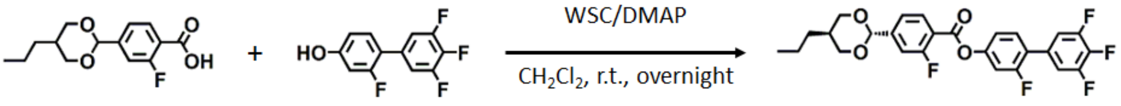

4

2-fluoro-4-(5-n-propyl-1,3-dioxan-2-yl)benzoic acid 2.15 g (8.00 mmol) and WSC 1.89 g (9.86 mmol) were dissolved in CH_2Cl_2 (15 mL) and then solution was stirred for 10 min. To this solution, 3-fluoro-4-(3,4,5-trifluorophenyl)phenol 2.32 g (9.60 mmol) and DMAP 50 mg (0.41 mmol) were added and the mixture was stirred at ambient temperature for 15 h. The solution was added in CH_2Cl_2 (80 mL) and then the organic layer was washed with distilled water (100 mL x 3) and then dried over anhydrous Na_2SO_4. After evaporation, the resulting solid was purified by recrystallization from CH_2Cl_2/MeOH to give colorless crystals in trans-form. Yield: 980 mg (24.9%). ^{1}H NMR (400 MHz, $CDCl_3$, ppm): δ 8.09 (t, *J* = 7.8 Hz, 2H; phenyl), 7.45-7.38 (m, 3H; phenyl), 7.20-7.13 (m, 4H; phenyl), 5.46 (s, 1H; acetal), 4.27 (dd, *J* = 12, 8.8 Hz, 2H; -O-CH_2-), 3.56 (t, *J* = 12 Hz, 2H; -O-CH_2-), 2.21-2.12 (m, 1H, -C$\underline{H}$-CH_2O-), 1.40-1.31 (m, 2H; CH_3-C$\underline{H}_2$-), 1.11 (q, *J* = 7.9 Hz, 2H; CH_3-CH_2-C$\underline{H}_2$-), 0.94 (t, *J* = 7.4 Hz, 3H, C$\underline{H}_3$-CH_2-). ^{13}C{^{1}H} NMR (100 MHz, $CDCl_3$, ppm): δ 162.0 (d, $^3J_{C-F}$ = 4 Hz, C=O), 162.4 (d, $^1J_{C-F}$ = 260 Hz, C-F in benzoate), 159.3 (d, $^1J_{C-F}$ = 249 Hz, C-F in phenol), 151.2 (ddd, $^1J_{C-F}$ = 248 Hz, $^2J_{C-F}$ = 10 Hz, $^3J_{C-F}$ = 4 Hz, C-F in trifluorophenyl), 151.3 (d, $^3J_{C-F}$ = 11 Hz, C-O in phenol), 146.7 (d, $^3J_{C-F}$ = 9 Hz, benzoate), 139.5 (dt, $^1J_{C-F}$ = 252 Hz, $^2J_{C-F}$ = 15 Hz, C-F in trifluorophenyl), 132.5 (s, benzoate), 130.9 (dt, $^3J_{C-F}$ = 12 Hz, $^4J_{C-F}$ = 4 Hz, trifluorophenyl), 130.6 (d, $^3J_{C-F}$ = 4 Hz, phenol), 124.2 (d, $^2J_{C-F}$ = 12 Hz, phenol), 121.9 (d, $^3J_{C-F}$ = 3 Hz, benzoate), 118.2 (d, $^4J_{C-F}$ = 4 Hz, phenol), 117.3 (d, $^2J_{C-F}$ = 10 Hz, benzoate), 115.2 (d, $^2J_{C-F}$ = 24 Hz, benzoate), 113.2 (ddd, $^2J_{C-F}$ = 16 Hz, $^3J_{C-F}$ = 7 Hz, $^4J_{C-F}$ = 3 Hz, trifluorophenyl), 110.7 (d, $^2J_{C-F}$ = 26 Hz, phenol), 99.4 (s, acetal), 77.6 (s,

C-O in dioxane), 33.9 (s, CH_3-CH_2-CH_2-C), 30.2 (s, CH_3-CH_2-CH_2), 19.5 (s, CH_3-CH_2), 14.2 (s, CH_3). ^{19}F NMR (376 MHz, $CDCl_3$, ppm): -108.6 (1H, benzoate), -115.8 (1H, phenol), -135.6 (2H, trifluorophenyl), -162.6 (1H, trifluorophenyl). HRMS (EI) *m/z*: [M^+] calcd for $C_{26}H_{21}F_5O_4$: 492.1360; found: 492.1357. Anal. calcd for $C_{26}H_{21}F_5O_4$: C 63.42, H 4.30; found: C 63.61, H 4.32.

1-4. Synthesis of 3-fluoro-4-(3,4,5-trifluorophenyl)phenyl 2-chloro-4-(trans-5-n-propyl-1,3-dioxan-2-yl)benzoate (5)

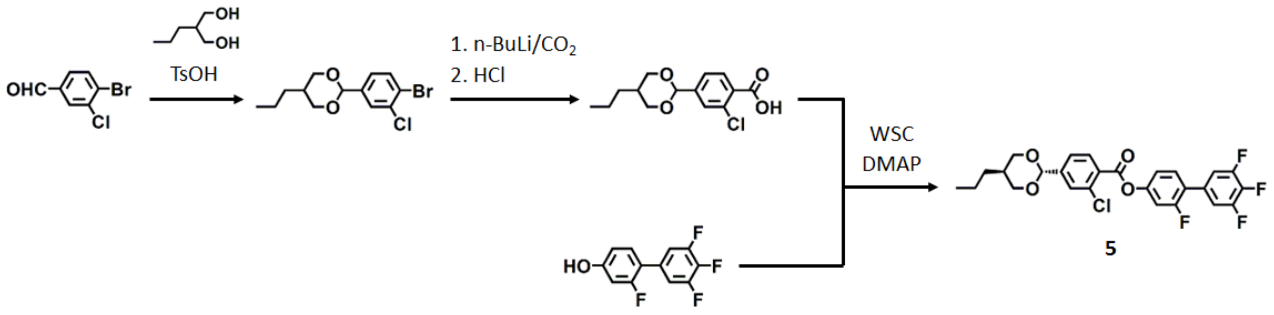

1-4-1. 1-Bromo-2-chloro-4-(5-n-propyl-1,3-dioxan-2-yl)benzene

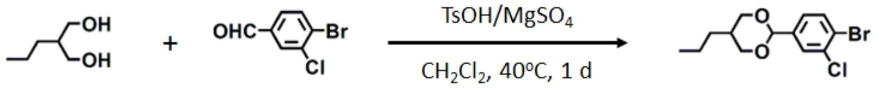

To CH_2Cl_2 (25 mL), 2-n-propyl-1,3-propanediol 3.23 g (27.3 mmol), 4-bromo-3-chlorobenzaldehyde 4.92 g (22.4 mmol) and TsOH· H_2O 438 mg (2.30 mmol) were dissolved. Anhydrous $MgSO_4$ 14.5 g (121 mmol) was added and the mixture was stirred at 40°C for 1 day. The mixture was poured into cold water (200 mL) containing $NaHCO_3$ 1.0 g (12 mmol) and oily fractions were extracted with CH_2Cl_2 (200 mL). The organic layer was washed with distilled water (200 mL x 3) and then dried with anhydrous Na_2SO_4. After evaporation and dryness under reduced pressure, pale orange liquids were obtained with a cis/trans ratio of 3:7. Yield: 7.10 g (97.4%). ^{1}H NMR (400 MHz, DMSO-d_6, ppm): δ 7.75 (s, 1H; phenyl), 7.58 (s, 1H; phenyl), 7.29 (s, 1H; phenyl), 5.52 (s, 0.3H; cis-form acetal), 5.43 (s, 0.7H; trans-form acetal), 4.14-4.11 (m, 1.4H; -CH_2-O- in trans-form), 4.05-3.92 (m, 1.2H; cis-form -CH_2-O-), 3.52-3.58 (m, 1.4H; -CH_2-O- in trans-form), 1.96-1.93 (m, 0.7H; -CH-CH_2O- in trans-form), 1.62 (m, 0.6H; CH_3-CH_2-CH_2- in cis-form), 1.42-1.25 (m, 2.4H; -CH-CH_2O- in cis-form, CH_3-CH_2-CH_2- in cis-form and CH_3-CH_2-CH_2- in trans-form), 1.02 (br, 1.4H, CH_3-CH_2-CH_2- in trans-form), 0.85 (m, 3H; CH_3-).

1-4-2. 2-Chloro-4-(5-n-propyl-1,3-dioxan-2-yl)benzoic acid

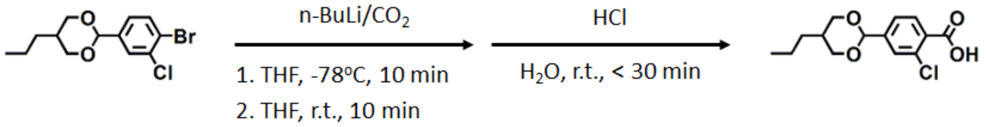

To dehydrated THF (20 mL) was added 1-bromo-2-chloro-4-(5-n-propyl-1,3-dioxan-2-yl)benzene 3.20 g (10.0 mmol) and then the solution was cooled to -78°C in a bath of dry ice/acetone. After 1.6 M n-butyl lithium hexane solution (7.5 mL, 12 mmol, 1.2 equiv.) was slowly added at -78°C, the solution was stirred at the temperature for 10 min. After that, dry ice 11 g (250 mmol) was added and the mixture was stirred at -78°C

for 10 min and then stirred at ambient temperature for 10 min. After an addition of H_2O (1 mL), the solvents were removed. The residues were dissolved in distilled water (200 mL) and washed with CH_2Cl_2 (100 mL x 3). The aqueous layer was collected and its pH was adjusted to 2-3 by an addition of 1 M HCl aq. The resulting precipitates were collected by suction filtration and washed thoroughly with water. After drying under reduced pressure, white solids with a cis/trans ratio of 3:7 were obtained. Yield: 2.01 g (69.7%). ^{1}H NMR (400 MHz, $CDCl_3$, ppm): δ 8.01 (d, *J* = 8.0 Hz, 1H; phenyl), 7.64 (s, 1H; phenyl), 7.46 (d, *J* = 9.6 Hz, 1H; phenyl), 5.51 (s, 0.3H; cis-form acetal), 5.42 (s, 0.7H; trans-form acetal), 4.25 (dd, *J* = 12, 4.4 Hz, 1.3H; -CH_2-O- in trans-form), 4.12-4.05 (m, 1.3H; CH_2-O- in cis-form), 3.54 (t, *J* = 11 Hz, 1.3H; -CH_2-O- in trans-form), 2.20-2.09 (m, 0.6H; -C<u>H</u>-CH_2O- in trans-form), 1.78-1.72 (m, 0.6H; CH_3-CH_2-C<u>H</u>$_2$- in cis-form), 1.49-1.30 (m, 2.2H; -C<u>H</u>-CH_2O- in cis-form, CH_3-C<u>H</u>$_2$-CH_2- in cis-form and CH_3-CH_2-C<u>H</u>$_2$- in trans-form), 1.09 (q, *J* = 7.7 Hz, 1.5H, CH_3-C<u>H</u>$_2$-CH_2- in trans-form), 0.98-0.91 (m, 3H; CH_3-).

1-4-3. 3-Fluoro-4-(3,4,5-trifluorophenyl)phenyl 2-chloro-4-(trans-5-n-propyl-1,3-dioxan-2-yl)benzoate (**5**)

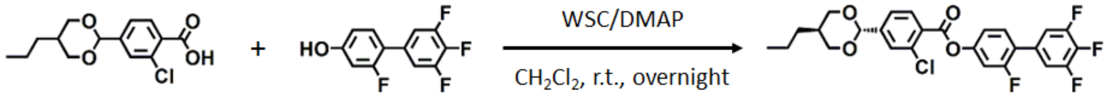

WSC 2.76 g (14.4 mmol) and 2-chloro-4-(5-n-propyl-1,3-dioxan-2-yl)benzoic acid 3.42 g (12.0 mmol) were dissolved in dehydrated CH_2Cl_2 (20 mL) and then solution was stirred at ambient temperature for 10 min. To the solution, 3-fluoro-4-(3,4,5-trifluorophenyl)phenol 3.49 g (14.4 mmol) and DMAP 73 mg (0.06 mmol) were added and the solution was stirred at rt. for 17 h. The solution was added in CH_2Cl_2 (100 mL) and washed with distilled water (100 mL x 4) and the organic layer was dried with anhydrous Na_2SO_4. After evaporation, orange residues were purified by silica-gel column chromatography (CH_2Cl_2/MeOH volume ratio of 50:1). The solids were further purified by successive recrystallization from MeOH to give pure trans-form as white solids. Yield: 530 mg (8.6%). ^{1}H NMR (400 MHz, $CDCl_3$, ppm): δ 8.05 (d, *J* = 7.6 Hz, 1H; phenyl), 7.69 (s, 1H; phenyl), 7.52 (d, *J* = 9.6 Hz, 1H; phenyl), 7.43 (t, *J* = 8.8 Hz, 1H; phenyl), 7.20-7.15 (m, 4H; phenyl), 5.44 (s, 1H; acetal), 4.26 (dd, *J* = 12, 5.0 Hz, 2H; -O-CH_2-), 3.56 (t, *J* = 12 Hz, 2H; -O-CH_2-), 2.21-2.12 (m, 1H, -C<u>H</u>-CH_2O-), 1.40-1.31 (m, 2H; CH_3-C<u>H</u>$_2$-), 1.11 (q, *J* = 7.6 Hz, 2H; CH_3-CH_2-C<u>H</u>$_2$-), 0.94 (t, *J* = 7.8 Hz, 3H, C<u>H</u>$_3$-CH_2-). ^{13}C{^{1}H} NMR (100 MHz, $CDCl_3$, ppm): δ 163.2 (s, C=O), 159.4 (d, $^1J_{C\text{-}F}$ = 250 Hz, C-F in phenol), 151.2 (ddd, $^1J_{C\text{-}F}$ = 248 Hz, $^2J_{C\text{-}F}$ = 10 Hz, $^3J_{C\text{-}F}$ = 4 Hz, C-F in trifluorophenyl), 151.3 (d, $^3J_{C\text{-}F}$ = 11 Hz, C-O in phenol), 144.3 (s, benzoate), 139.5 (dt, $^1J_{C\text{-}F}$ = 251 Hz, $^2J_{C\text{-}F}$ = 15 Hz, C-F in trifluorophenyl), 134.7 (s, C-Cl in benzoate), 132.0 (s, benzoate), 130.9 (dt, $^3J_{C\text{-}F}$ = 13 Hz, $^4J_{C\text{-}F}$ = 3 Hz, trifluorophenyl), 130.6 (d, $^3J_{C\text{-}F}$ = 4 Hz, phenol), 129.4 (s, benzoate), 128.4 (s, benzoate), 124.6 (s, benzoate), 124.1 (d, $^2J_{C\text{-}F}$ = 12 Hz, phenol), 118.2 (d, $^4J_{C\text{-}F}$ = 4 Hz, phenol), 113.2 (ddd, $^2J_{C\text{-}F}$ = 15 Hz, $^3J_{C\text{-}F}$ = 7 Hz, $^4J_{C\text{-}F}$ = 3 Hz, trifluorophenyl), 110.7 (d, $^2J_{C\text{-}F}$ = 26 Hz, phenol), 99.5 (s, acetal), 77.6 (s, C-O in dioxane), 33.9 (s, CH_3-CH_2-CH_2-<u>C</u>), 30.3 (s, CH_3-CH_2-<u>C</u>H_2), 19.5 (s, CH_3-<u>C</u>H_2), 14.2 (s, CH_3). ^{19}F NMR (376 MHz, $CDCl_3$, ppm): -115.7 (1H, phenol), 135.5 (2H, trifluorophenyl), -162.5 (1H, trifluorophenyl). HRMS (EI) *m*/*z*: [M$^+$] calcd for $C_{26}H_{21}ClF_4O_4$: 508.1065; found: 508.1065. Anal. calcd for $C_{26}H_{21}ClF_4O_4$: C 61.36, H 4.16; found: C 61.36, H 4.19.

1-5. Synthesis of 3-fluoro-4-(3,4,5-trifluorophenyl)phenyl 4-(trans-5-n-propyl-1,3-dioxan-2-yl)benzoate (6)

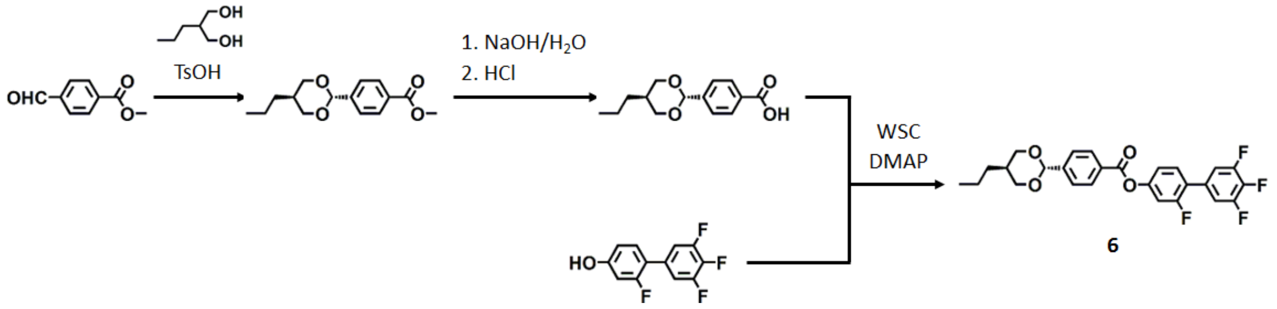

6

1-5-1. 4-(trans-5-n-propyl-1,3-dioxan-2-yl)benzoic acid methyl ester

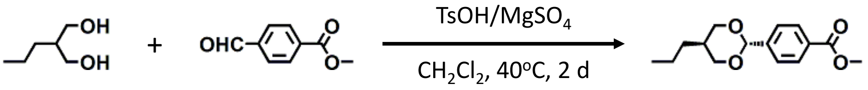

To a CH_2Cl_2 solution (20 mL) of 2-n-propyl-1,3-propanediol 1.42 g (12.0 mmol) and methyl terephthalaldehydate 1.64 g (10.0 mmol) were added TsOH· H_2O 190 mg (1.00 mmol) and anhydrous $MgSO_4$ 12.0 g (100 mmol). The mixture was stirred at 40°C for 2 days and then added into cold distilled water (100 mL). After extraction with CH_2Cl_2 (200 mL), the organic layer was washed with distilled water (200 mL x 3) and then dried over anhydrous Na_2SO_4. After concentration, the resulting solids (crude yield, 2.58 g) was purified by recrystallization from MeOH. White solids with trans-form were obtained. Yield: 1.17 g (44.3%). ^{1}H NMR (400 MHz, DMSO-d_6, ppm): δ 7.96 (d, *J* = 8.8 Hz, 2H; phenyl), 7.55 (d, *J* = 7.6 Hz, 2H; phenyl), 5.51 (s, 1H; acetal), 4.15 (dd, *J* = 12, 4 Hz, 2H; m, 2H: -O-CH_2-), 3.54 (t, *J* = 12 Hz, 2H: -O-CH_2-), 3.86 (s, 3H; -$COOCH_3$), 1.99-1.95 (m, 1H; -C$\underline{H}$-CH_2O-), 1.34-1.24 (m, 2H, CH_3-C$\underline{H}_2$-), 1.08-1.02 (m, 2H; CH_3-CH_2-C$\underline{H}_2$-), 0.88 (t, *J* = 7.4 Hz, 3H, C$\underline{H}_3$-CH_2-).

1-5-2. 4-(trans-5-n-propyl-1,3-dioxan-2-yl)benzoic acid

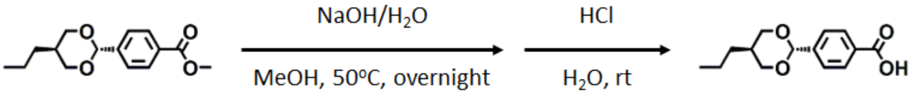

To a methanol solution (30 mL) of 4-(trans-5-propyl-1,3-dioxan-2-yl)benzoic acid methyl ester 1.17 g (5.00 mmol) was added an aqueous solution (5.0 mL) of NaOH 2.0 g (50 mmol). The mixture was stirred at 50°C for 13 h and then concentrated by rotary evaporator. The residue was dissolved in distilled water (50 mL) and the pH of the solution was adjusted to 5 by an addition of 1M HCl aq. The precipitates was collected by suction filtration and then washed with distilled water. After drying under reduced pressure, trans-4-(5-n-propyl-1,3-dioxan-2-yl)benzoic acid was obtained as a white solid. Yield: 1.07 g (85.3%). ^{1}H NMR (400 MHz, DMSO-d_6, ppm): δ 13.0 (br, 1H: COOH), 7.94 (d, *J* = 8.0 Hz, 2H; phenyl), 7.52 (d, *J* = 8.8 Hz, 2H; phenyl), 5.51 (s, 1H; acetal), 4.15 (dd, *J* = 12, 4.8 Hz, 2H; -O-CH_2-) 3.54 (t, *J* = 11 Hz, 2H; -O-CH_2-), 2.02-1.93 (m, 1H; -C$\underline{H}$-CH_2O-), 1.34-1.24 (m, 2H, CH_3-C$\underline{H}_2$-), 1.08-1.02 (m, 2H; CH_3-CH_2-C$\underline{H}_2$-), 0.88 (t, *J* = 7.4 Hz, 3H, C$\underline{H}_3$-CH_2-).

1-5-3. 3-Fluoro-4-(3,4,5-trifluorophenyl)phenyl 4-(trans-5-n-propyl-1,3-dioxan-2-yl)benzoate (**6**)

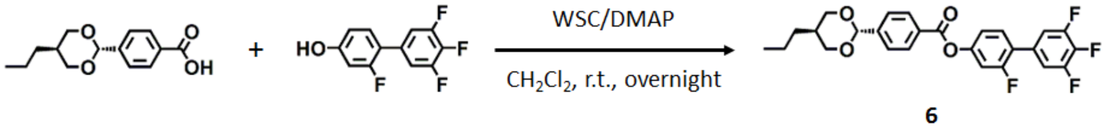

WSC 1.99 g (10.4 mmol) and 4-(trans-5-n-propyl-1,3-dioxan-2-yl)benzoic acid 2.00 g (7.99 mmol) were dissolved in CH_2Cl_2 (20 mL) and the solution was stirred at ambient temperature for 30 min. To the solution, 3-fluoro-4-(3,4,5-trifluorophenyl)phenol 2.52 g (10.4 mmol) and DMAP 98 mg (0.80 mmol) were added and then the solution stirred at rt. for 18 h. The solution was added to CH_2Cl_2 (50 mL) and washed with distilled water (50 mL x 3) and then the organic layer dried over Na_2SO_4. After evaporation, the residue was purified by reprecipitation from MeOH to give crude products (2.65 g, 70.0%). The solids were further purified by silica-gel column chromatography and recrystallization from MeOH/$CHCl_3$ at 4°C to afford white solids. Yield: 2.09 g (55.1%). ^{1}H NMR (400 MHz, DMSO-d_6, ppm): δ 8.14 (d, J = 8.8 Hz, 2H; phenyl), 7.71-7.60 (m, 5H; phenyl), 7.49 (dd, J = 11, 2.0 Hz, 1H; phenyl), 7.32 (dd, J = 8.4, 2.6 Hz, 1H; phenyl), 5.57 (s, 1H; acetal), 4.17 (dd, J = 12, 4.8 Hz, 2H; -O-CH_2-), 3.56 (t, J = 11 Hz, 2H; -O-CH_2-), 2.05-1.96 (m, 1H, -C$\underline{H}$-CH_2O-), 1.35-1.25 (m, 2H; CH_3-C$\underline{H}_2$-), 1.06 (q, J = 7.9 Hz, 2H; CH_3-CH_2-C$\underline{H}_2$-), 0.88 (t, J = 7.4 Hz, 3H, C$\underline{H}_3$-CH_2-). ^{13}C{^{1}H} NMR (100 MHz, $CDCl_3$, ppm): δ 164.4 (s, C=O), 159.4 (d, $^1J_{C-F}$ = 249 Hz, C-F in phenol), 151.7 (d, $^3J_{C-F}$ = 12 Hz, C-O in phenol), 151.1 (ddd, $^1J_{C-F}$ = 248 Hz, $^2J_{C-F}$ = 11 Hz, $^3J_{C-F}$ = 5 Hz, C-F in trifluorophenyl), 144.2 (s, benzoate), 139.5 (dt, $^1J_{C-F}$ = 251 Hz, $^2J_{C-F}$ = 15 Hz, C-F in trifluorophenyl), 130.9 (dt, $^3J_{C-F}$ = 12 Hz, $^4J_{C-F}$ = 4 Hz, trifluorophenyl), 130.6 (d, $^3J_{C-F}$ = 4 Hz, phenol), 130.2 (s, benzoate), 129.0 (s, benzoate), 126.5 (s, benzoate), 123.8 (d, $^2J_{C-F}$ = 13 Hz, phenol), 118.2 (d, $^4J_{C-F}$ = 4 Hz, phenol), 113.2 (ddd, $^2J_{C-F}$ = 15 Hz, $^3J_{C-F}$ = 6 Hz, $^4J_{C-F}$ = 3 Hz, trifluorophenyl), 110.7 (d, $^2J_{C-F}$ = 26 Hz, phenol), 100.5 (s, acetal), 72.7 (s, C-O in dioxane), 34.0 (s, CH_3-CH_2-CH_2-$\underline{C}$), 30.3 (s, CH_3-CH_2-$\underline{C}H_2$), 19.5 (s, CH_3-$\underline{C}H_2$), 14.2 (s, CH_3). ^{19}F NMR (376 MHz, $CDCl_3$, ppm): -115.8 (1H, phenol), 135.6 (2H, trifluorophenyl), -162.6 (1H, trifluorophenyl). HRMS (EI) m/z: [M^+] calcd for $C_{26}H_{22}F_4O_4$: 474.1454; found: 474.1454. Anal. calcd for $C_{26}H_{22}F_4O_4$: C 65.82, H 4.67; found: C 65.88, H 4.63.

SI-2. DSC charts of compounds 2-6

a. compound 2

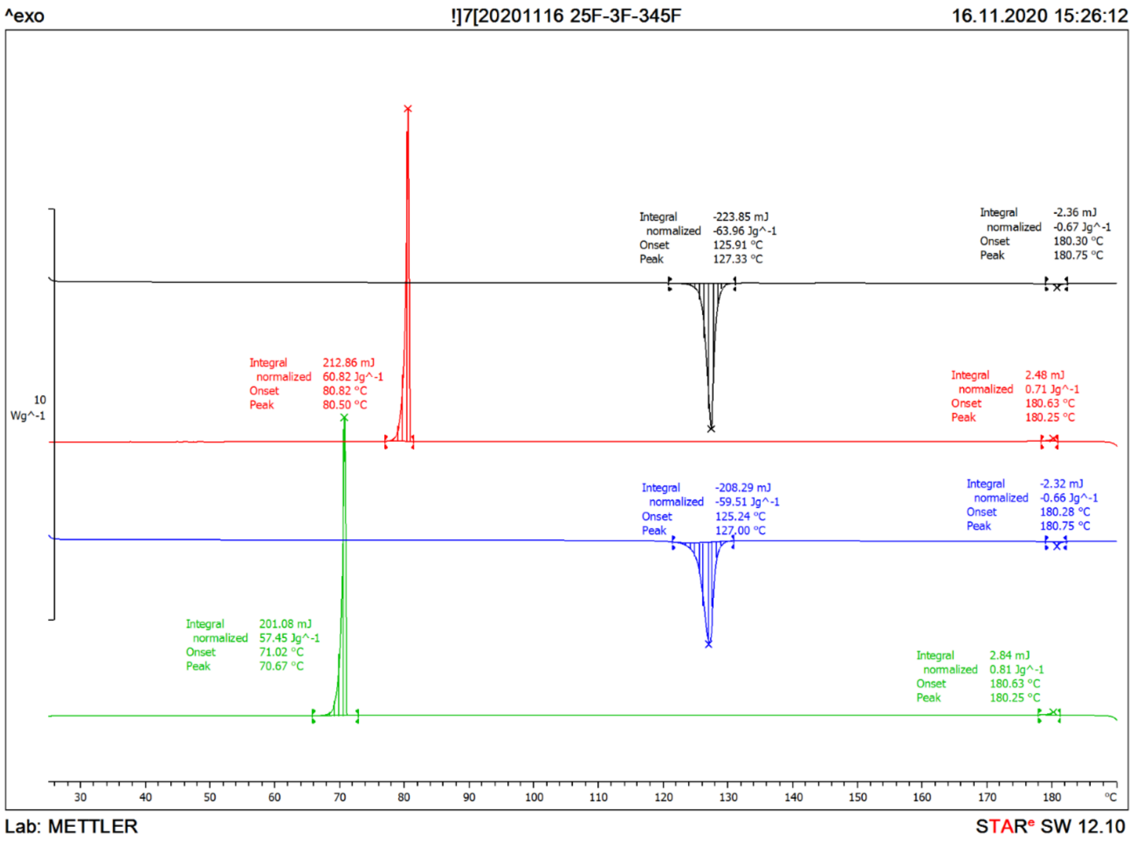

b. compound 3

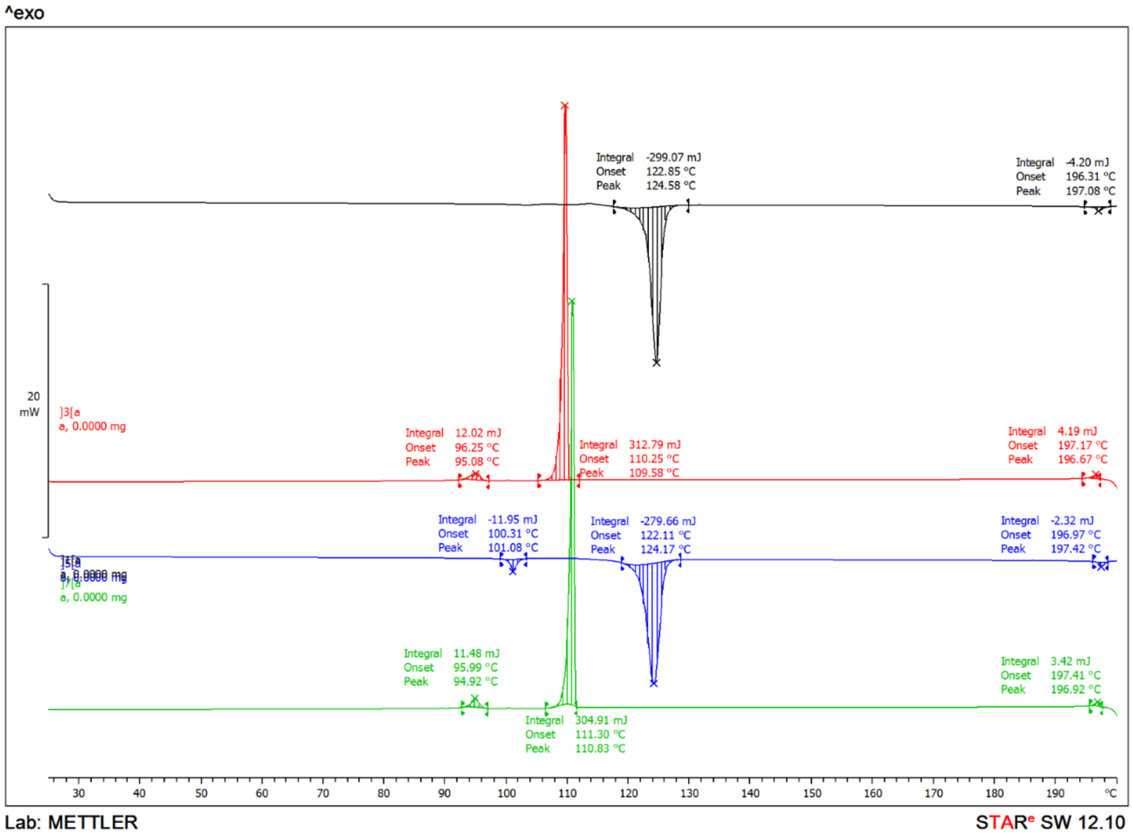

c. compound 4

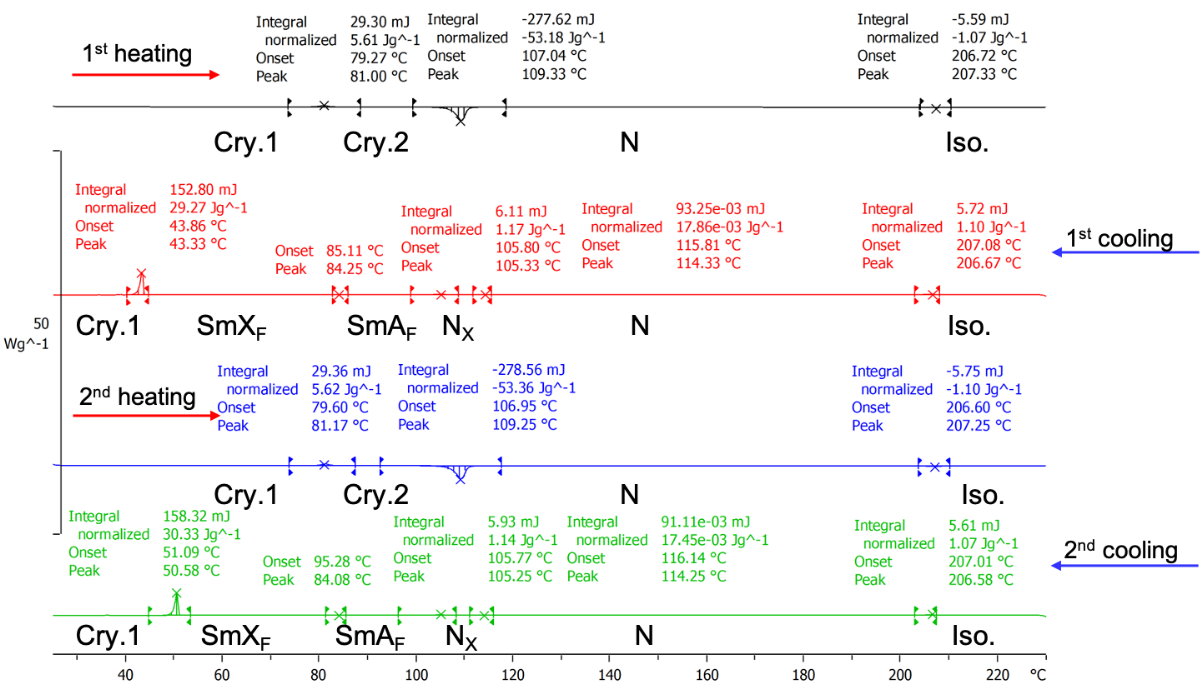

d. compound 4

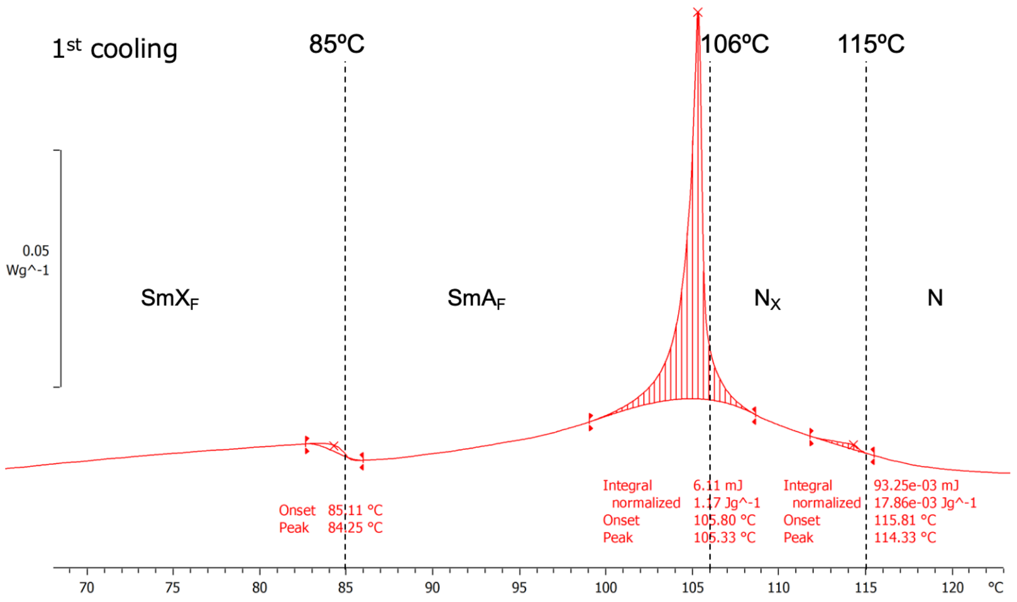

e. compound 5

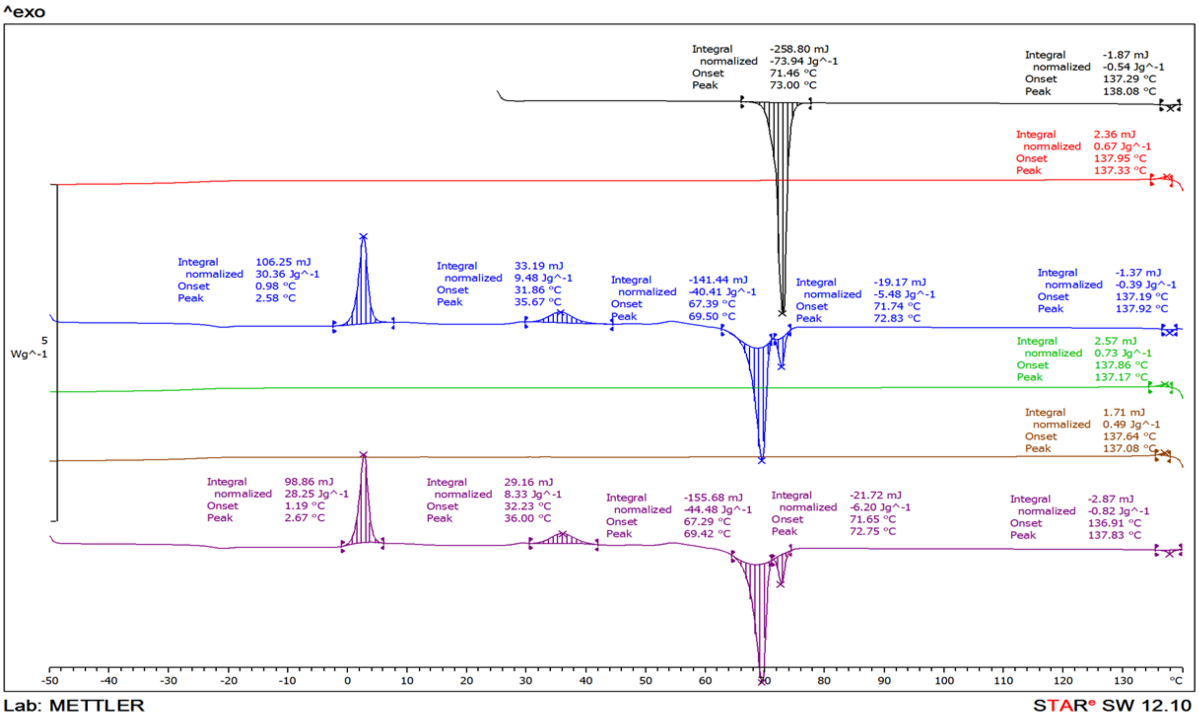

f. compound 6

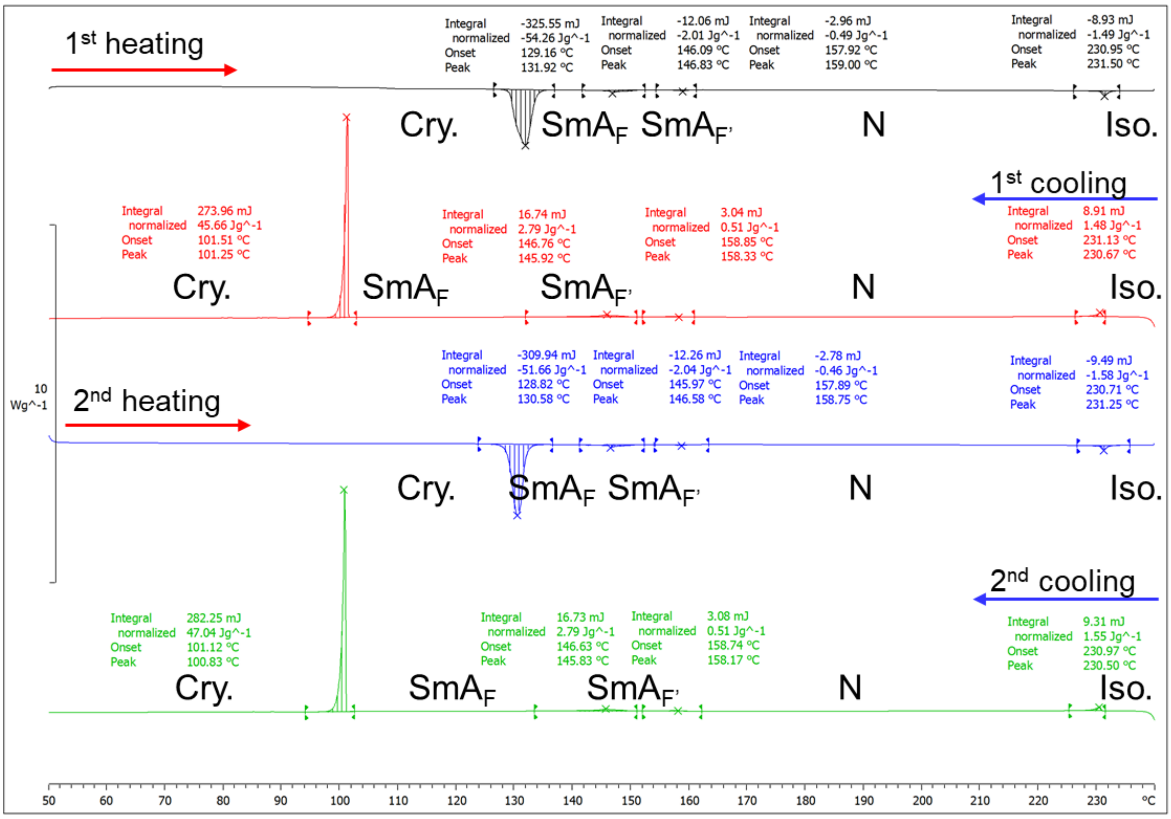

g. compound 6

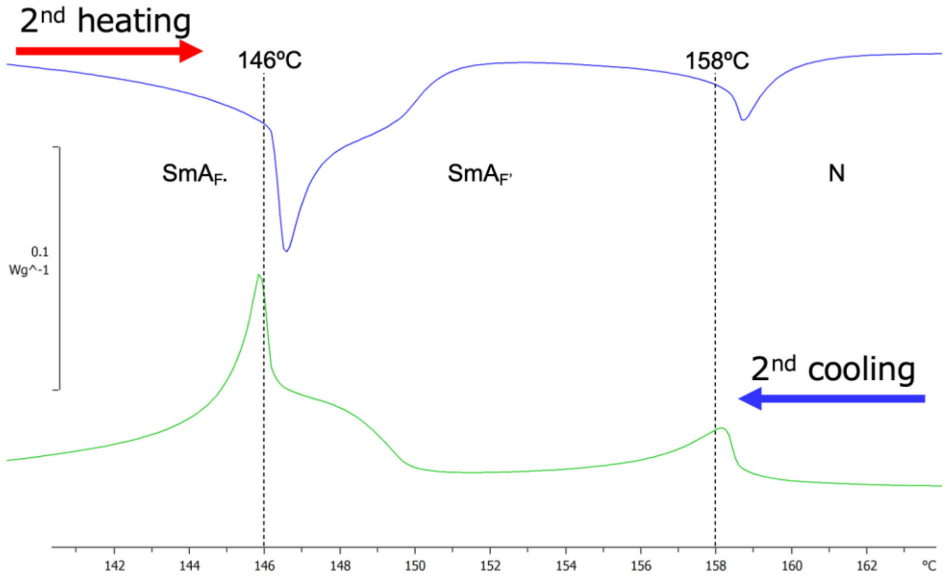

Figure S2. DSC charts of compounds, a)**2**, b)**3**, c)**4**, e)**5** and f)**6**, d) enlarged view of chart c, and g) enlarged view of chart f.

DSC measurements were performed at a heating and cooling rate of 5 °C min^{-1}.

SI-3. Polarizing optical scope observations

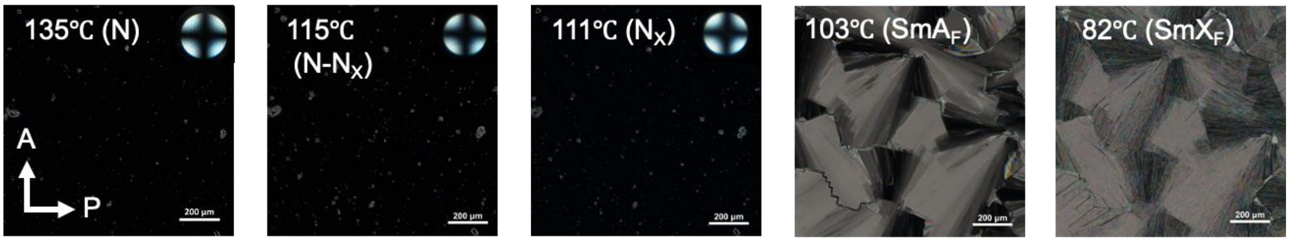

Figure S3-1. Temperature dependence of polarized optical microscopy images of compound **4** using cells with vertical alignment treatment. The crossed dark lines image in the upper right corner of the picture is isogyre by conoscopic observation, indicating a homeotropic orientation. The scale bar in each image represents 200 μm.

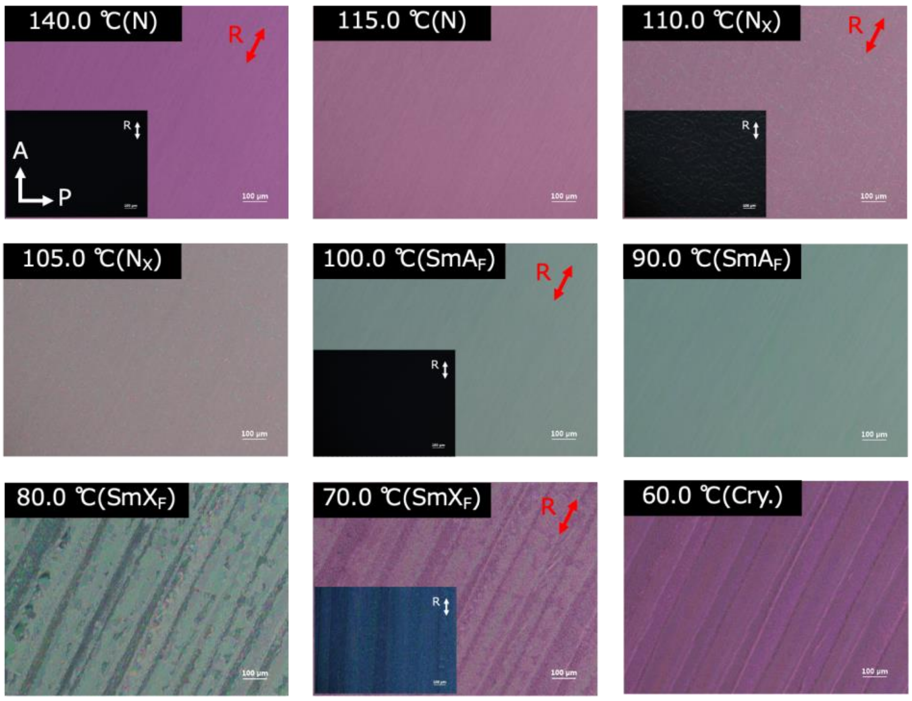

Figure S3-2. Temperature dependence of polarized optical microscopy images of compound **4** using cells with rubbing orientation treatment. The arrow R represents the rubbing direction, and A and P represent the orientation of the analyzer and polarizer of the polarizing optical microscope. In the N, N_X, and SmA_F phases, the extinction position appeared when the rubbing direction coincided with the A or B polarization axis, indicating that the director is oriented parallel to the rubbing direction, which is a homogeneous orientation. The scale bar in each image represents 100 μm.

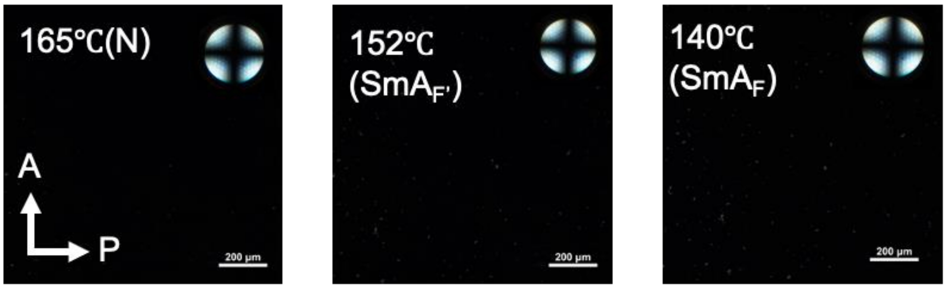

Figure S3-3. Temperature dependence of polarized optical microscopy images of compound **6** using cells with vertical alignment treatment. The crossed dark lines image in the upper right corner of the picture is isogyre by conoscopic observation, indicating a homeotropic orientation. The scale bar in each image represents 200 µm.

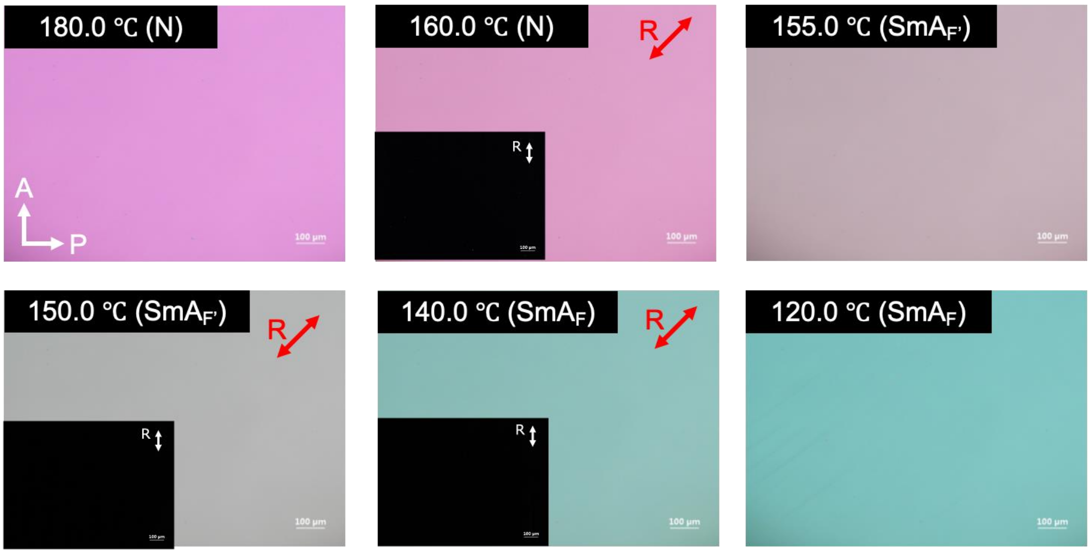

Figure S3-4. Temperature dependence of polarized optical microscopy images of compound **6** using cells with rubbing orientation treatment. The arrow R represents the rubbing direction, and A and P represent the orientation of the analyzer and polarizer of the polarizing optical microscope. In the N, SmA$_{F'}$, and SmA$_F$ phases, the extinction position appeared when the rubbing direction coincided with the A or B polarization axis, indicating that the director is oriented parallel to the rubbing direction, which is a homogeneous orientation. The scale bar in each image represents 100 µm.

SI-4. Dielectric permittivities of compounds 2, 3 and 5

a. compound **2**

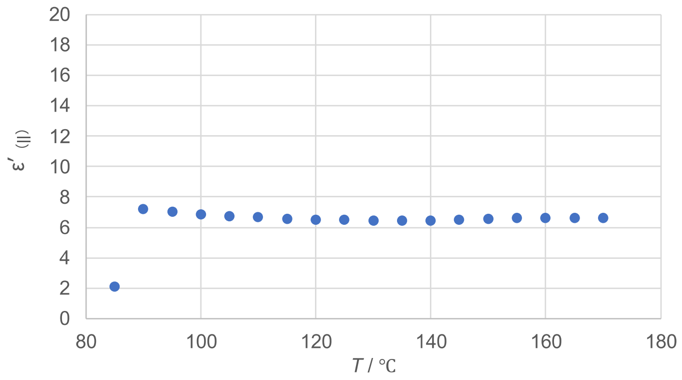

b. compound **3**

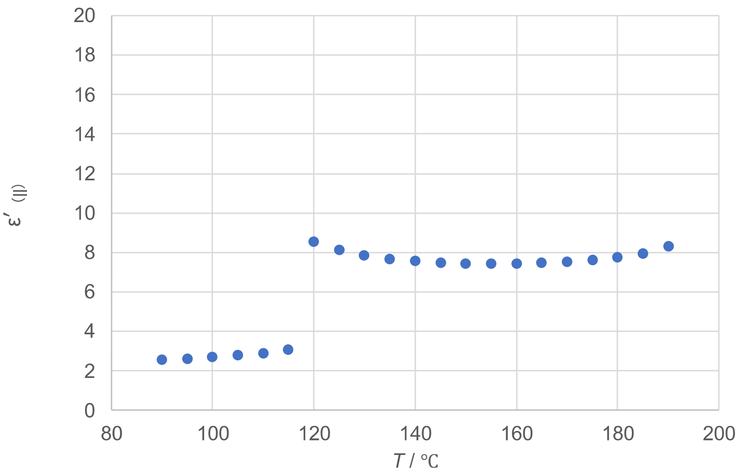

c. compound **5**

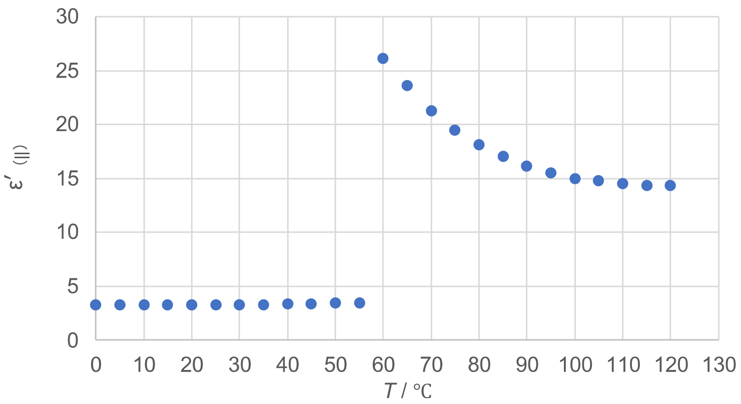

Figure S4-1. Temperature dependence of dielectric permittivity of compounds, a)**2** at 1 kHz, b)**3** at 10 kHz and c) **5** at 100 Hz. ε'(∥) is the dielectric permittivity in the direction parallel to the director, and was measured using a cell fabricated from substrates with transparent indium tin oxide (ITO) electrodes with vertically oriented surface treatment. The discontinuous drop in dielectric permittivity reflects crystallization of the sample.

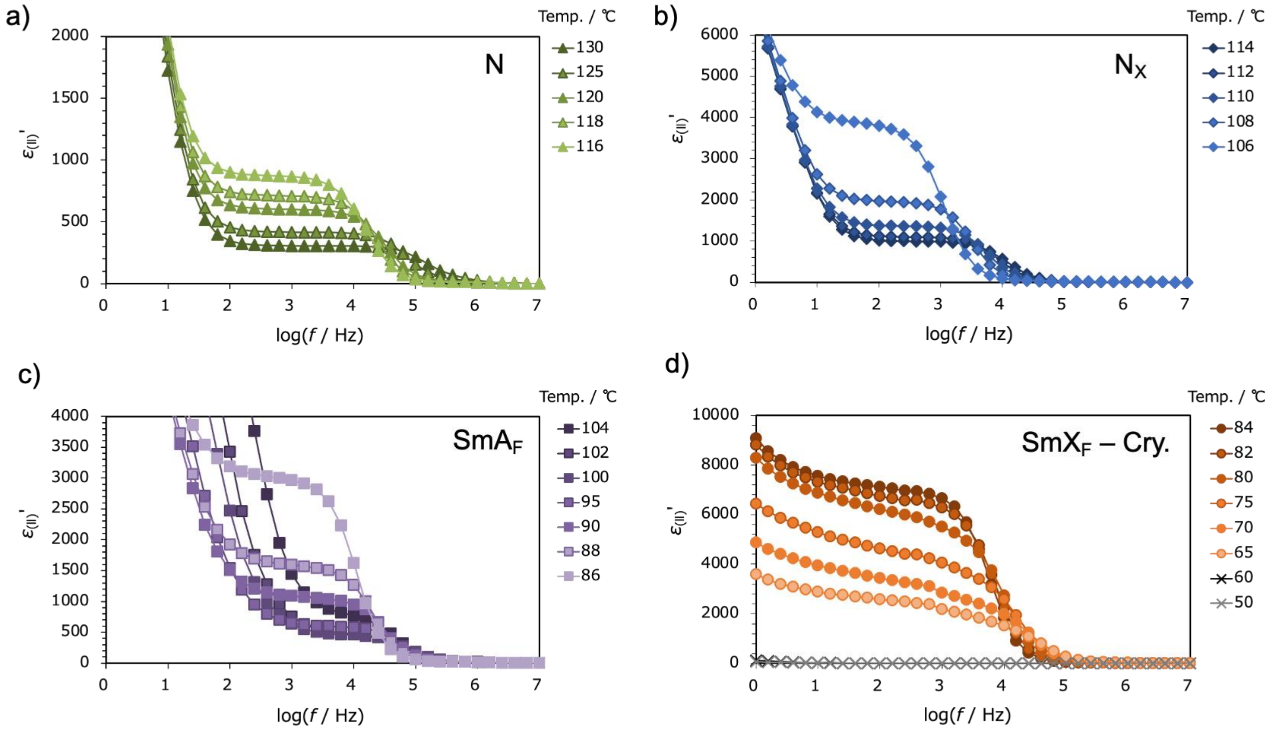

Figure S4-2. Frequency dispersion of the dielectric permittivity of compound **4** at various temperatures.

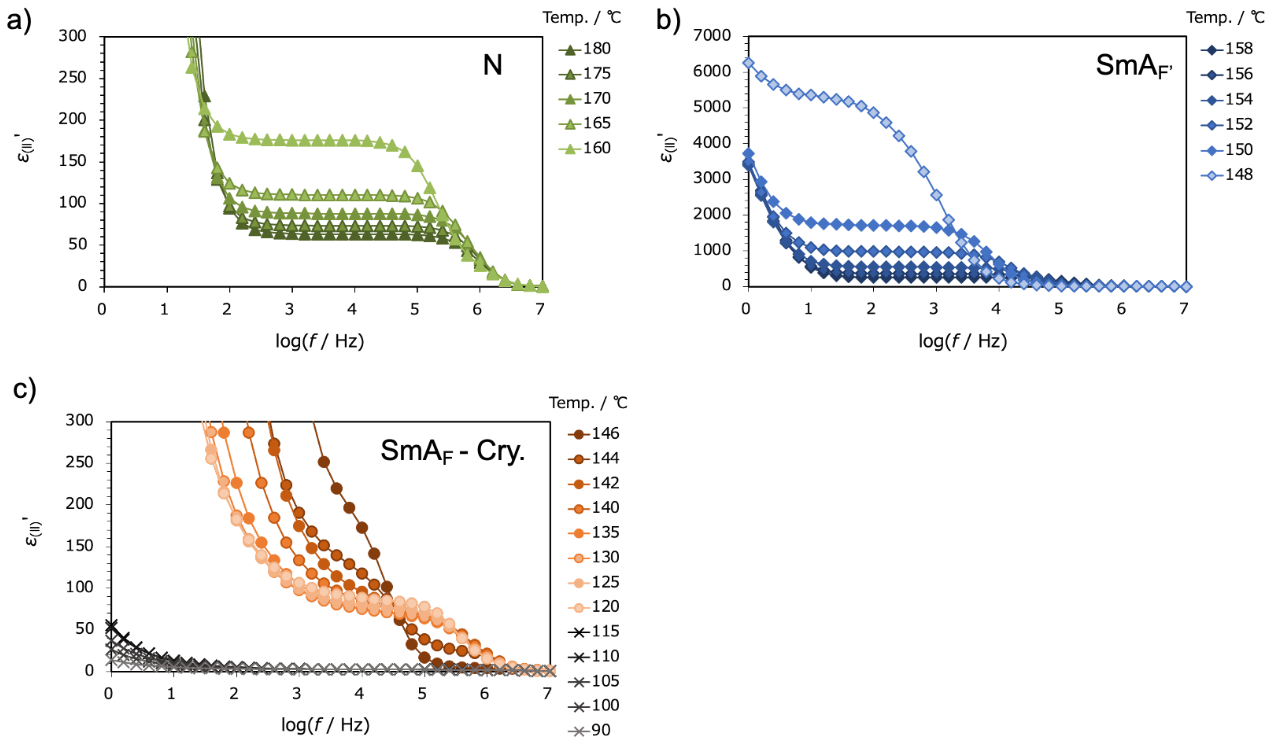

Figure S4-3. Frequency dispersion of the dielectric permittivity of compound **6** at various temperatures.

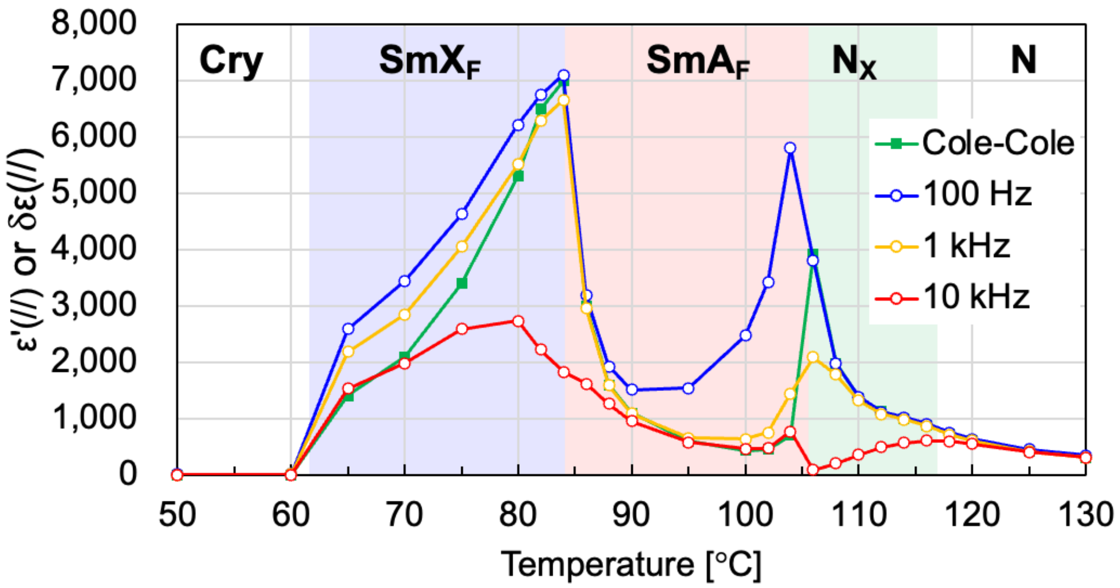

Figure S4-4. Temperature dependence of dielectric permittivity at various frequencies and relaxation strength (Cole-Cole) for compound **4**.

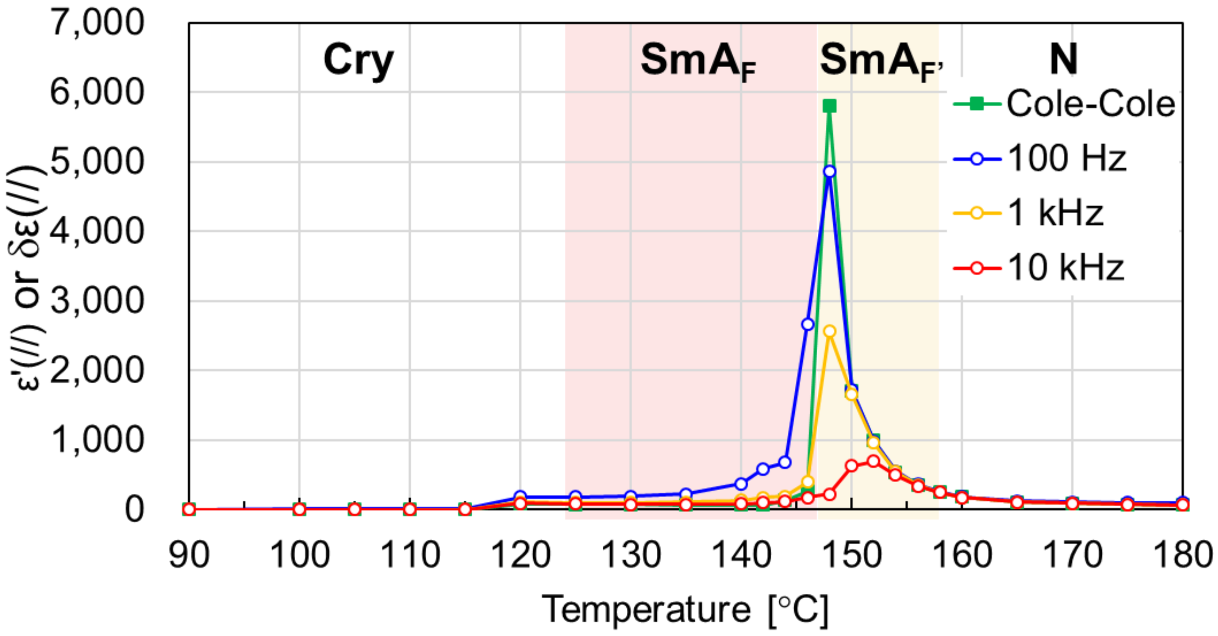

Figure S4-5. Temperature dependence of dielectric permittivity at various frequencies and relaxation strength (Cole-Cole) for compound **6**.

SI-5. Wide angle and small angle X-ray diffraction (WAXD) of 4 and 6

a.

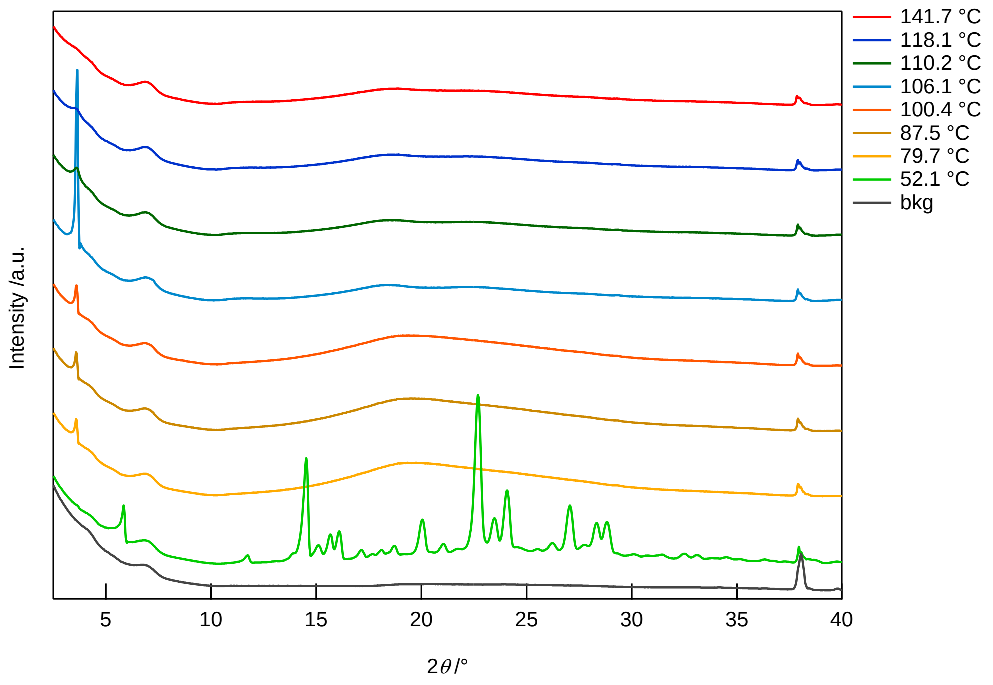

b.

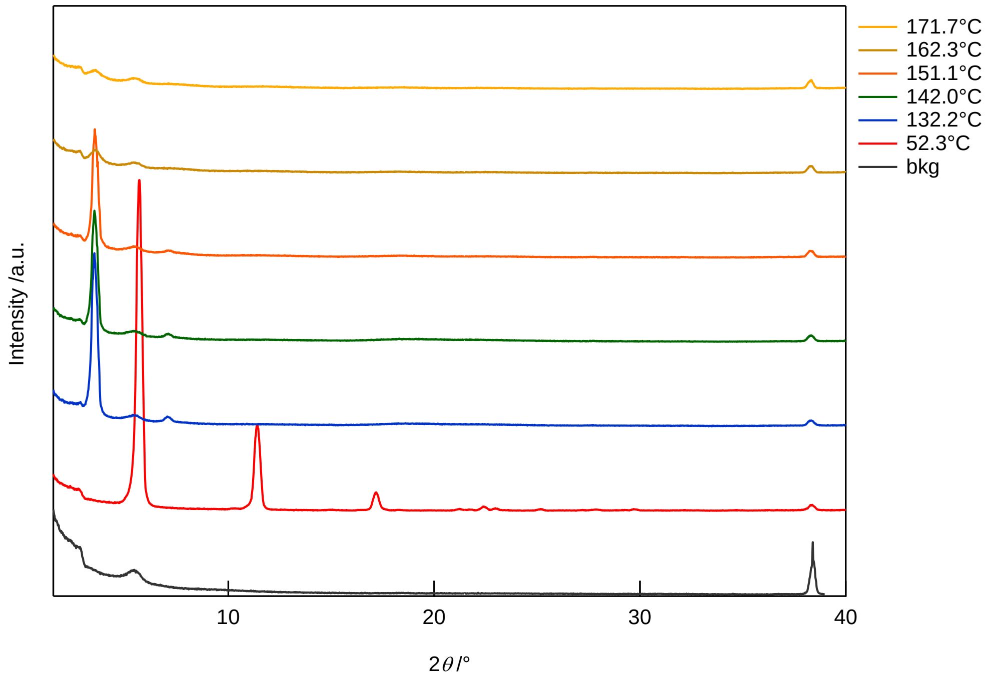

Figure S5-1. Wide angle X-ray diffraction profiles of compounds a)**4** and b)**6**.

a) The sharp peak around 3–4° is due to the layer structure of the smectic phase, and the broad diffuse scattering around 20° represents the liquid-like arrangement of molecules in the layer. There are peaks at angles around 4°, 7°, and 38° that do not originate from the sample.

b) The sharp peak around 3–4° is due to the layer structure of the smectic phase. There are peaks at angles around 5.4° and 38° that do not originate from the sample.

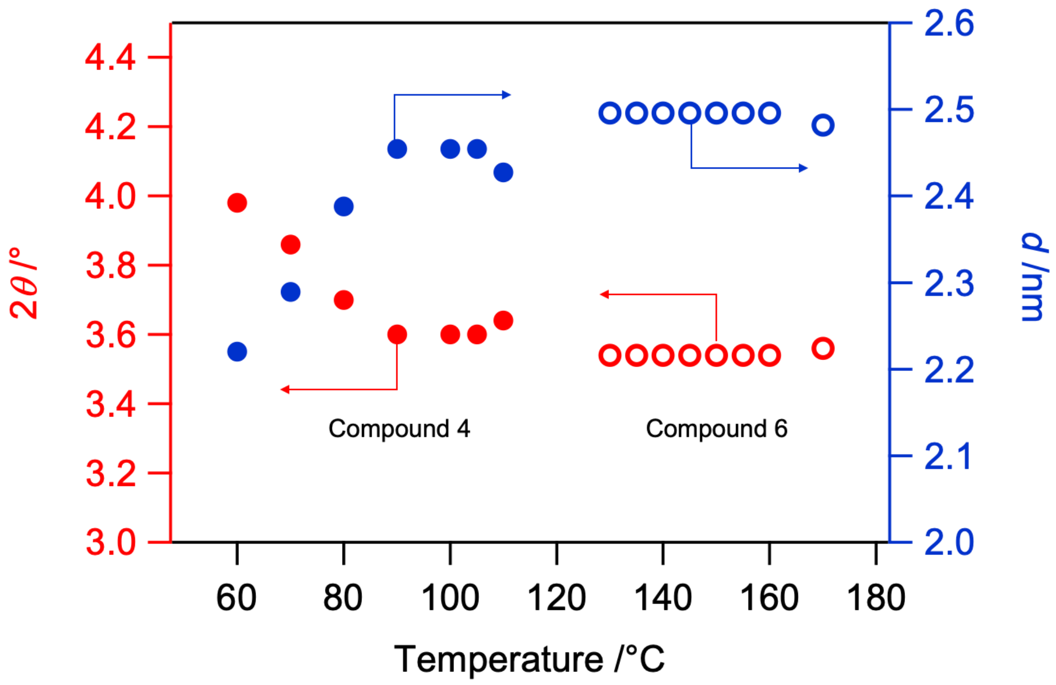

Figure S5-2. Temperature dependence of Bragg angle (left axis) and d-spacing (right axis) in compounds **4** (filled circle) and **6** (open circle).

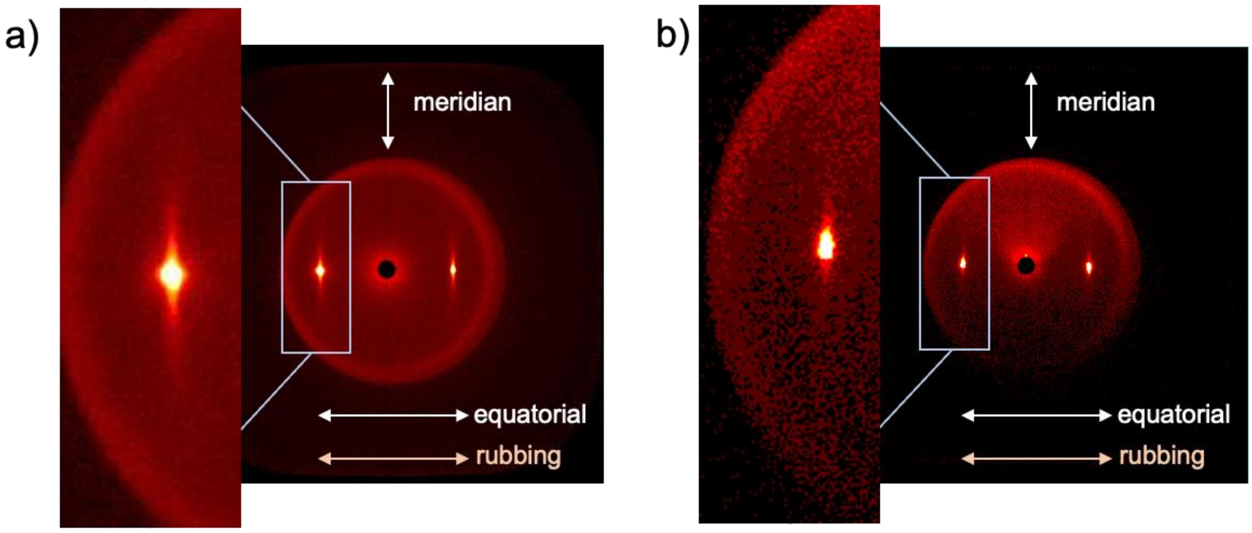

Figure S5-3. 2D SAXD images of compounds **4**(a) and **6**(b) in SmA_F and their magnified views

SI-6. Molecular length

Compound **4**

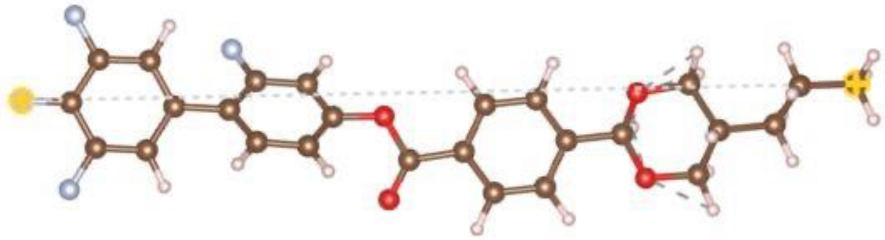

2.254 nm

Compound **6**

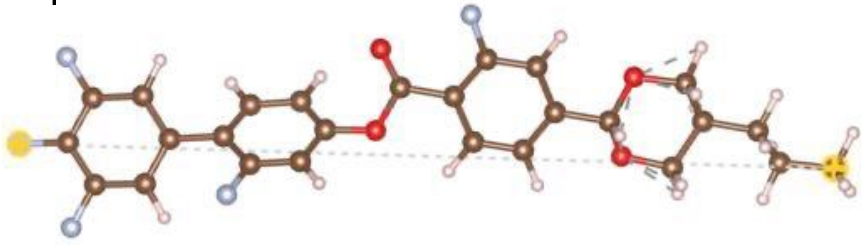

2.248 nm

Figure S6. Molecular structures and the molecular lengths of compound **4** and **6**. These structures were drawn by using VESTA 3.4.0.[1]

SI-7. Polarizing optical microscopy images of the compound 6 upon electric field on and off

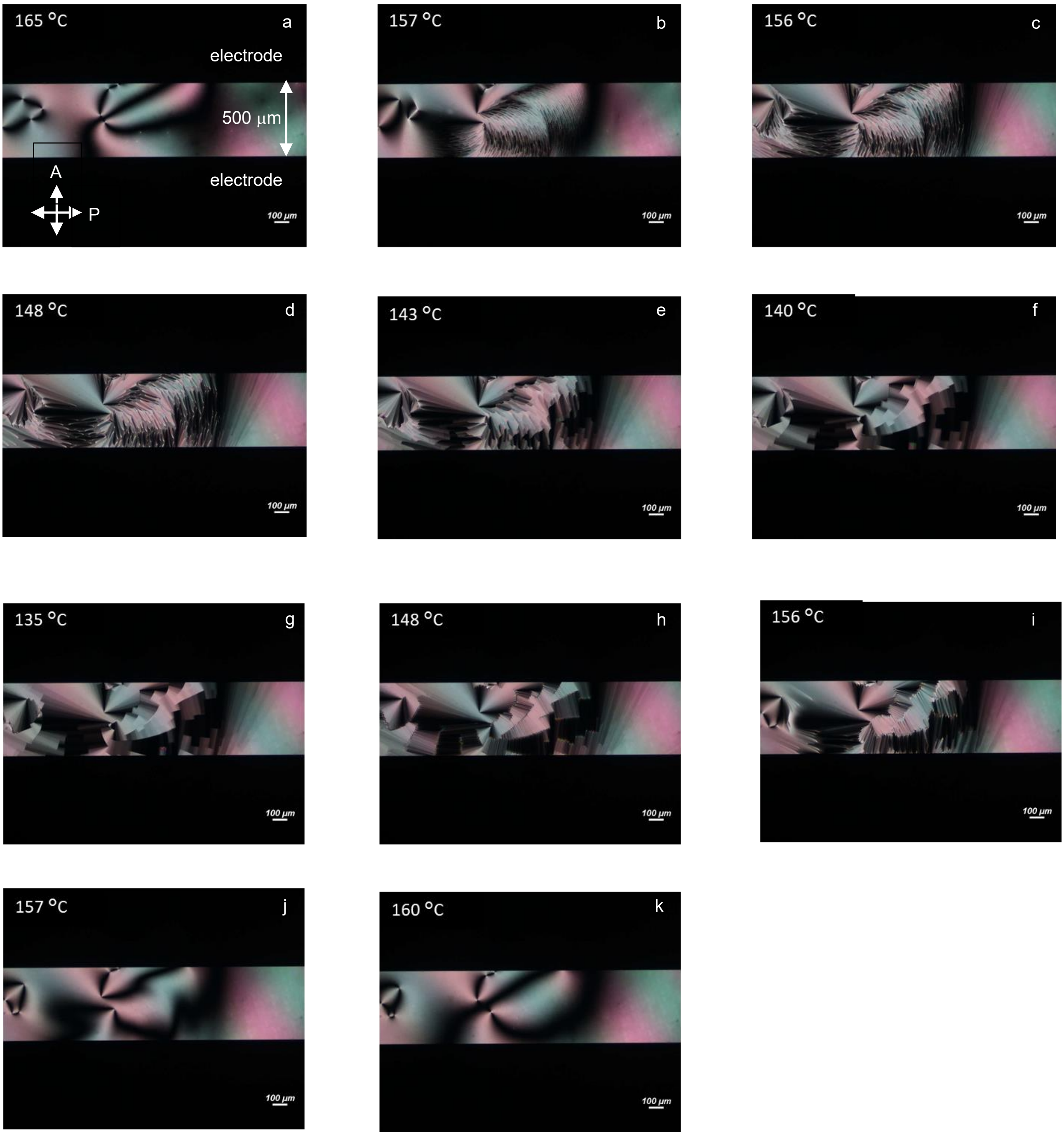

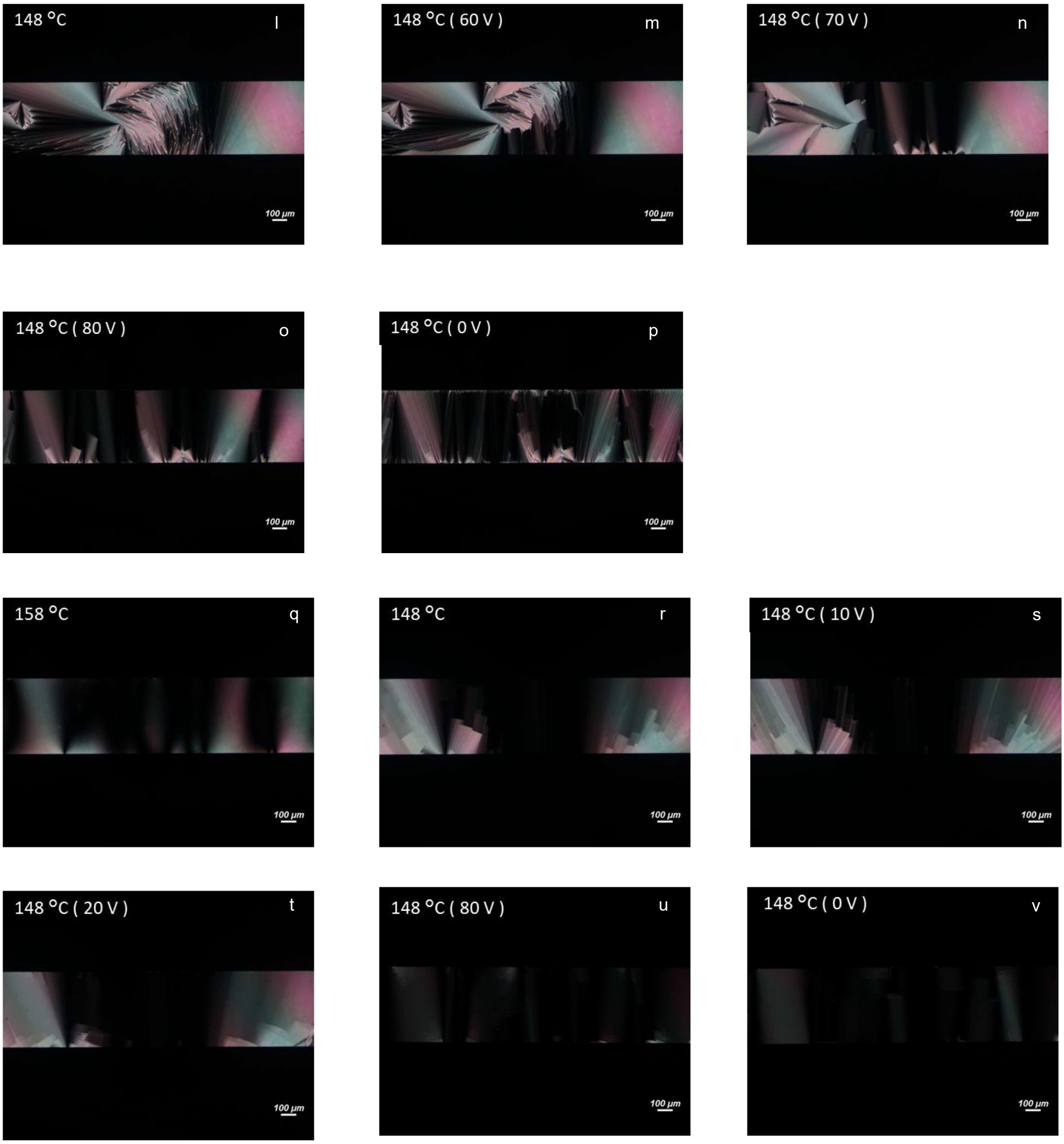

Figure S7. POM observation images of compound **6** upon off and on voltages.
The sample is contained in a cell with a substrate equipped with electrodes that can apply an electric field in the plane. The distance between electrodes was 500 μm. From a to g, the sample was cooled from the normal N phase to the SmA$_{F'}$ phase, and heat to N again from h to k. Texture was almost back to its original state. From i to v, after repeating the process of applying a voltage of about 80 V and turning it off twice (from p to r, the sample was once heated to N phase and cooled again to 148°C), the texture became almost uniformly oriented in the direction of the electric field, and this state was maintained stably even after the electric field was removed. The scale bar in each image represents 100 µm.

SI-8. SHG interference

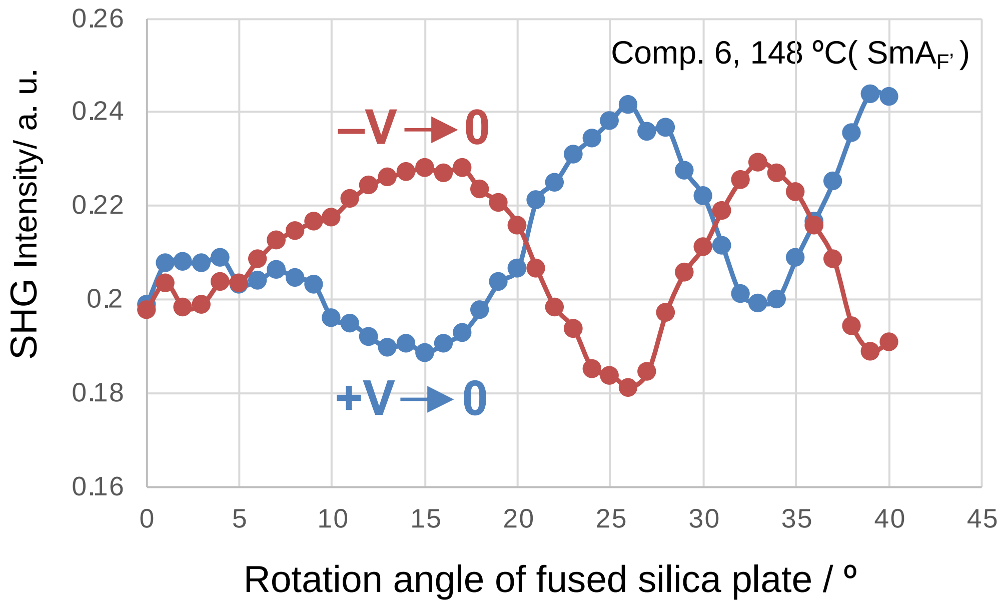

Figure S8. SHG fringe patterns of **6** in the SmA$_{F'}$ phase at 148°C under after removal of applied voltage.

Applied voltage was 80 V between electrodes with 500 μm gap. The horizontal axis of the graph is the rotation angle of the fused silica plate placed on the optical axis of the laser, which shifts the optical phase of the SHG light from the Y-cut quartz in front of it and interferes with the SHG light from the sample, causing the intensity to change periodically.

SI-9. Curie–Weiss plot

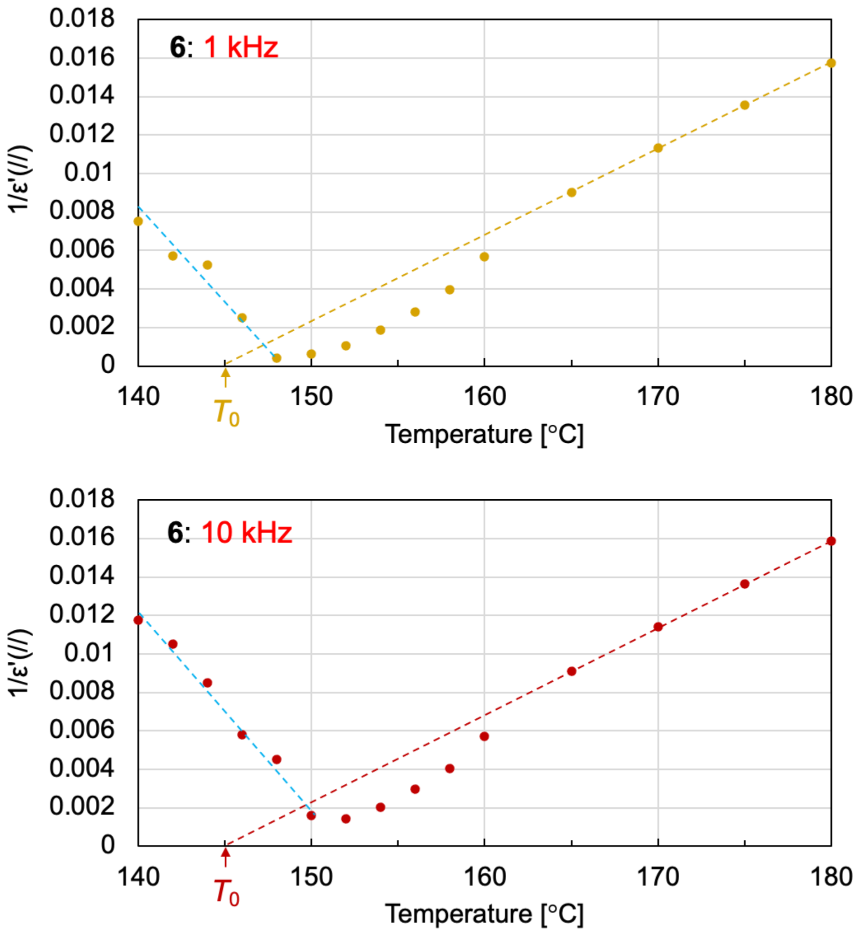

Figure S9. Relationship between temperature and the reciprocal of dielectric permittivity at 1 kHz and 10 kHz for compound **6**.

SI-10. Focal conic structure in a smectic phase

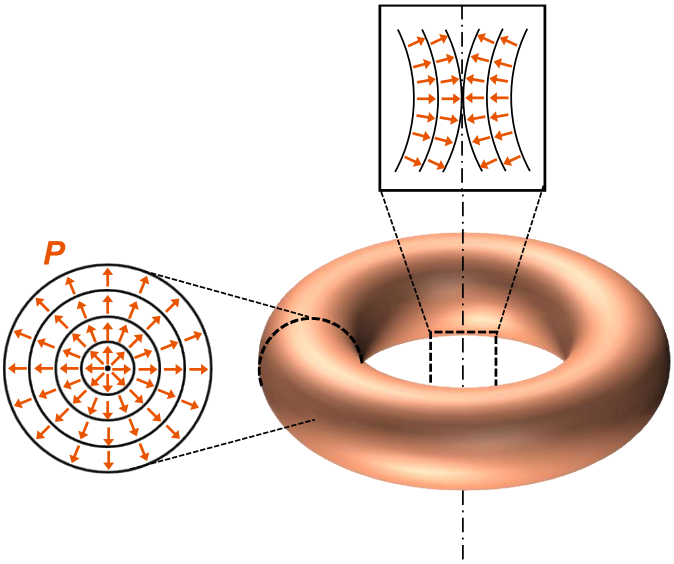

Figure S10. Schematic illustration of ferroelectric dipole alignment in layered tori.

If the P_S occurs along the layer normal in the layered tori and Dupin cyclide structures, the polarity of the spontaneous polarization ***P*** will be focused (div***P*** < 0) or diverged (div***P*** > 0) and opposed on the centerlines of the tube and torus, which is extremely disadvantageous electrostatically. Therefore, tori and Dupin cyclide structures are forbidden in the SmA_F phase, and the layers are mainly arranged in parallel. Thus, focal conic and fan-shaped textures are not formed, and angular mosaic textures are preferentially induced.

1) Momma, K. & Izumi, F. VESTA 3 for three-dimensional visualization of crystal, volumetric and morphology data *Journal of Applied Crystallography* **44**, 1272–1276 (2011).
